# Biomimetic Adaptive Hydrothermal Balance Fabric‐Based Dual‐Interface Solar Evaporator for Efficient and Stable Desalination

**DOI:** 10.1002/advs.202521275

**Published:** 2025-12-12

**Authors:** Ning Niu, Lingjie Yu, Jiaguang Meng, Wei Fan, Kaili Chen, Wanwan He, Yongzhen Wang, Yaming Liu, Ying Li, Zhaoling Sun, Chao Zhi

**Affiliations:** ^1^ School of Textile Science and Engineering Xi'an Polytechnic University Xi'an Shaanxi 710048 China; ^2^ Key Laboratory of Functional Textile Material and Product Ministry of Education Xi'an Polytechnic University Xi'an Shaanxi 710048 China; ^3^ Shaanxi Textile Science Research Institute Co., Ltd. Xi'an Shaanxi 710016 China; ^4^ College of Textile Science and Engineering Jiangnan University Wuxi Jiangsu 214122 China

**Keywords:** hydrothermal equilibrium, interfacial solar steam generator, octopus‐inspired structure, seawater desalination, spacer fabric

## Abstract

Matching energy input to water supply is key to efficient solar‐driven interfacial water evaporation, but conventional interfacial solar steam generators (ISSGs) fail to adapt to diurnal solar flux fluctuations, thus hindering the achievement of dynamic hydrothermal balance. Inspired by marine octopuses, a fabric‐based dual‐interface solar evaporator (PDMS‐CFs‐CFF‐SF) is developed by integrating 3D knitting and electrostatic flocking, enabling adaptation to light intensity changes. When light intensity exceeds the upper interface's water supply capacity and heat accumulates, hydrophobic spacer yarns facilitate the directional transfer of excess heat to the lower interface, thereby triggering dual‐interface evaporation. The lower interface, leveraging its large‐pore structure, regulates moisture content to match the transferred excess heat. Moreover, the octopus‐inspired structure increases the evaporation area, enriches vapor escape channels, enhances thermal insulation performance, and adapts to sunlight incident at different angles. Under 1 kW m^−2^ irradiation, the evaporator achieves an evaporation rate of 3.14 kg m^−2^ h^−1^ and an efficiency of 129.32%. This work provides a novel structural strategy for developing ISSGs with dynamic hydrothermal balance capabilities.

## Graphical abstract

This study combines the biological structure of octopuses with the “3D structure” of spacer fabrics to fabricate a dual‐interface solar evaporator with adaptive hydrothermal balance. Water adaptation is achieved through the differentiated design of the upper and lower layers, and dynamic hydrothermal balance is realized by dynamically regulating the heat distribution with spacer yarns.

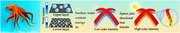

## 1 Introduction

With population growth, environmental pollution, and climate change, the world is confronting a severe shortage of freshwater resources.^[^ ^1^, ^2^, ^3^ ^]^ Seawater desalination, leveraging the advantage of abundant raw materials, stands as one of the ideal solutions to address this critical freshwater scarcity.^[^ ^4^, ^5^, ^6^ ^]^ Among various seawater desalination technologies, thermal‐based multi‐stage flash and membrane‐based reverse osmosis have matured.^[^ ^7^, ^8^, ^9^, ^10^ ^]^ However, their high energy consumption and substantial investment requirements have constrained their further application.^[^ ^11^, ^12^, ^13^ ^]^ Solar evaporation technology employs solar steam generators to convert absorbed solar energy into thermal energy for steam production.^[^ ^14^, ^15^ ^]^ Owing to its low energy consumption and sustainability, The solar evaporation technology is regarded as one of the most promising seawater desalination technologies.^[^ ^16^ ^]^ Nevertheless, significant thermal energy losses and low production efficiency have severely limited the further promotion and application of this technology.^[^ ^17^ ^]^ In contrast to traditional solar evaporation technology, solar‐driven interfacial evaporation technology utilizes an interfacial solar steam generator (ISSG) floating at the air/water interface to absorb solar radiation and convert it into thermal energy.^[^ ^18^, ^19^ ^]^ This technology enables the localization of solar‐generated thermal energy, thereby achieving efficient water distillation, and has emerged as a promising and sustainable approach to obtain freshwater from seawater or wastewater.^[^ ^20^, ^21^ ^]^ Furthermore, this fundamental process can be extended to various applications, including power generation,^[^ ^22^, ^23^, ^24^ ^]^ steam sterilization,^[^ ^25^, ^26^ ^]^ and fuel production.^[^ ^27^ ^]^

To enhance the steam generation performance of ISSGs, optimization from multiple dimensions is necessary, including improving solar energy absorption efficiency, reducing heat loss, suppressing salt scaling, and increasing water production capacity.^[^ ^28^, ^29^ ^]^ Toward this goal, exploring efficient and rational ISSG structures is an urgent priority. To date, a diverse range of ISSG structures have been developed, including umbrella‐shape,^[^ ^30^, ^31^ ^]^ arch‐shape,^[^ ^32^, ^33^ ^]^ mushroom‐shaped,^[^ ^34^, ^35^, ^36^ ^]^ and leaf‐shaped designs.^[^ ^37^, ^38^ ^]^ Through ingenious structural engineering, ISSGs have achieved substantial progress in enhancing evaporation performance.

However, kinetic mismatches in heat‐water transfer can lead to thermal‐hydraulic imbalance, which significantly constrains the stability and energy efficiency of ISSGs.^[^ ^39^ ^]^ Specifically, if solar energy input to the evaporator is substantially lower than water supply, heat is lost via thermal conduction as water flows into the seawater;^[^ ^40^, ^41^ ^]^ conversely, if solar energy input is significantly stronger than water supply, heat loss from thermal radiation and convection increases markedly, while the “dry spot” phenomenon at the gas‐liquid interface may induce salt crystallization and reduce light absorption efficiency.^[^ ^42^, ^43^ ^]^ Most previous efforts have focused on enhancing the evaporation rate and efficiency of ISSGs under specific light conditions (typically 1 kW m^−2^). In practical scenarios, however, solar radiation flux varies continuously across time and space.^[^ ^44^ ^]^ Some researchers have recognized this issue and addressed it through structural design to achieve dynamic hydrothermal balance, enabling efficient evaporation of ISSGs under varying solar intensities. For instance, Wei et al.^[^ ^45^ ^]^ proposed an integrated hydrophilic/hydrophobic Janus evaporator, which establishes an adaptive balance between solar thermal input and water absorption via unique unidirectional water transport induced by asymmetric wettability. Experimental and simulation results indicate that as light intensity increases, the water supply rate accelerates due to the dynamic management characteristics of water replenishment. Therefore, under 1 kW m^−2^ solar irradiation, the evaporation rate reaches up to 2.14 kg m^−2^ h^−1^, with an evaporation efficiency as high as 93.7%. Xiao et al.^[^ ^46^ ^]^ utilized centrifugal spinning to fabricate scalable, flexible, and stable photothermal evaporators using hydrophilic (polyacrylonitrile, PAN) and hydrophobic (polyurethane, PU) polymers. The combination of hydrophilic PAN and hydrophobic PU regulates water transport and supply to achieve thermal equilibrium, thereby reducing heat loss and promoting efficient evaporation. Under 1 kW m^−2^ irradiation with 10% saline water, the evaporator achieved a photothermal evaporation rate of 1.79 kg m^−2^ h^−1^. These studies highlight the importance of water‐thermal balance for enhancing evaporator performance from a water management perspective. Nevertheless, based on the unilateral management of either water or heat, challenges remain in achieving ISSGs with water‐heat synergistic management capabilities and thus a comprehensive dynamic water‐heat balance.

**Figure 1:**
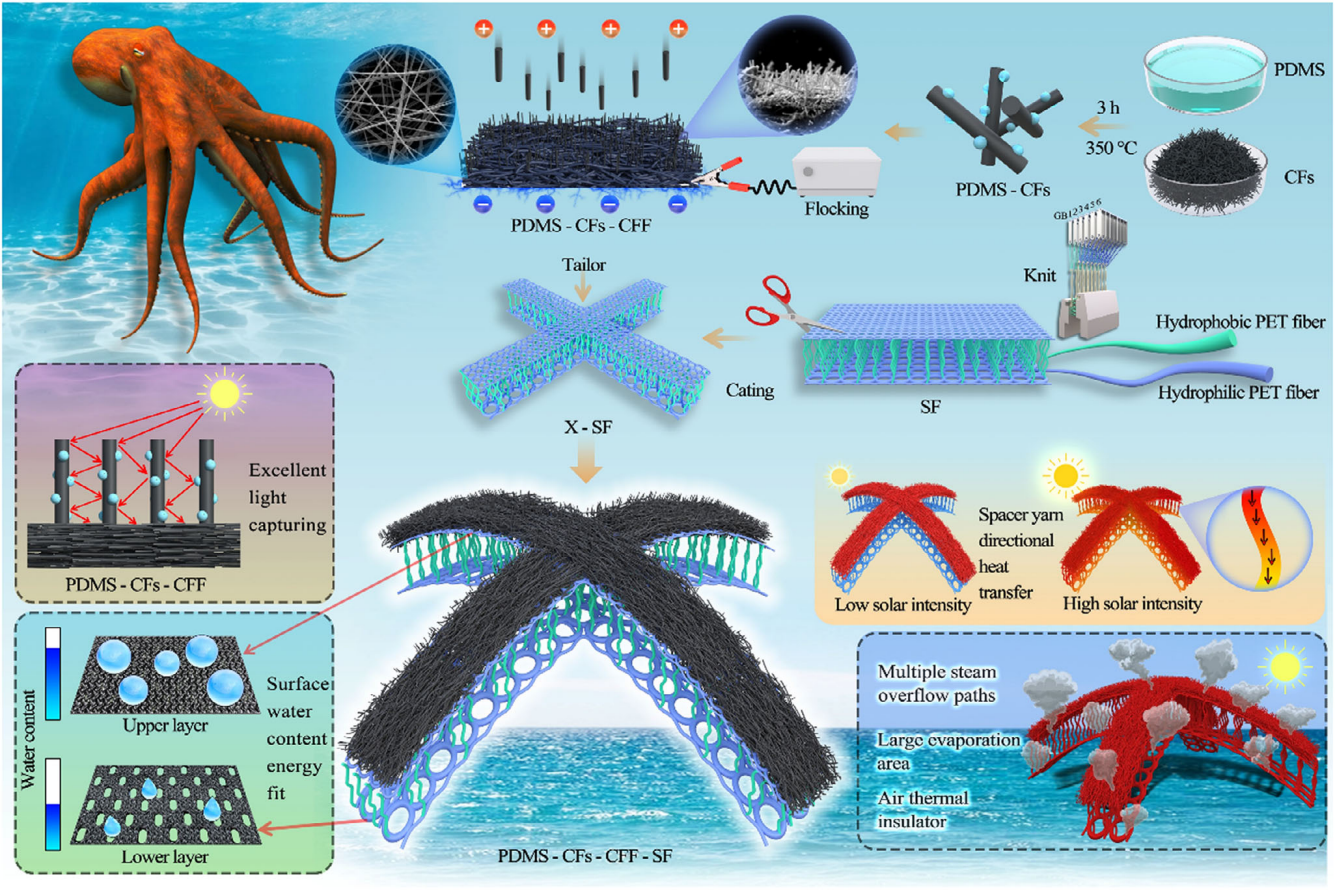
Preparation process and mechanism schematic of PDMS‐CFs‐CFF‐SF.

Based on the above considerations, inspired by the unique structure of octopuses in the ocean, we developed a fabric‐based dual‐interface solar evaporator (PDMS‐CFs‐CFF‐SF) with a dynamic hydrothermal synergistic management function. Its fabrication process and key characteristics are illustrated in **Figure** **1**. First, a warp‐knitted spacer fabric (SF) with a special structure was prepared using 3D knitting technology. Subsequently, the SF was cut into a bionic “X” shape mimicking an octopus. Next, carbon fibers (CFs) were subjected to simple thermal treatment to obtain superhydrophobic carbon fibers (PDMS‐CFs), which were then deposited on the carbon fiber felt (CFF) surface via electrostatic flocking to construct a superhydrophobic photothermal layer (PDMS‐CFs‐CFF). Finally, the PDMS‐CFs‐CFF was combined with the upper layer of the SF, resulting in the fabrication of a novel high‐performance ISSG. The photothermal layer of this evaporator has a special 3D structure, which enables multiple reflections and scattering of light to improve light absorption efficiency. The spacer yarn layer can dynamically regulate heat distribution and channel excess heat from the upper interface to the lower interface for dual‐interface evaporation, thereby enhancing energy utilization efficiency. The distinctive large‐pore structure of the lower interface allows it to regulate its own water content to match the available heat supply. Additionally, the evaporator's octopus‐inspired structure not only provides a greater evaporation area, more steam escape channels, and superior thermal insulation performance but also facilitates height adjustment to adapt to sunlight incident at different angles. Benefiting from this excellent structural design, the PDMS‐CFs‐CFF‐SF evaporator achieved a high evaporation rate of 3.14 kg m^−2^ h^−1^ (efficiency: 129.32%) under 1 kW m^−2^ illumination, surpassing most ISSGs using CFs as photothermal materials. In summary, this study provides new insights into advancing the hydrothermal balance design of ISSGs.

## 2 Results and Discussion

### 2.1 Morphology and Chemical Structure of PDMS‐CFs‐CFF‐SF

**Figure 2:**
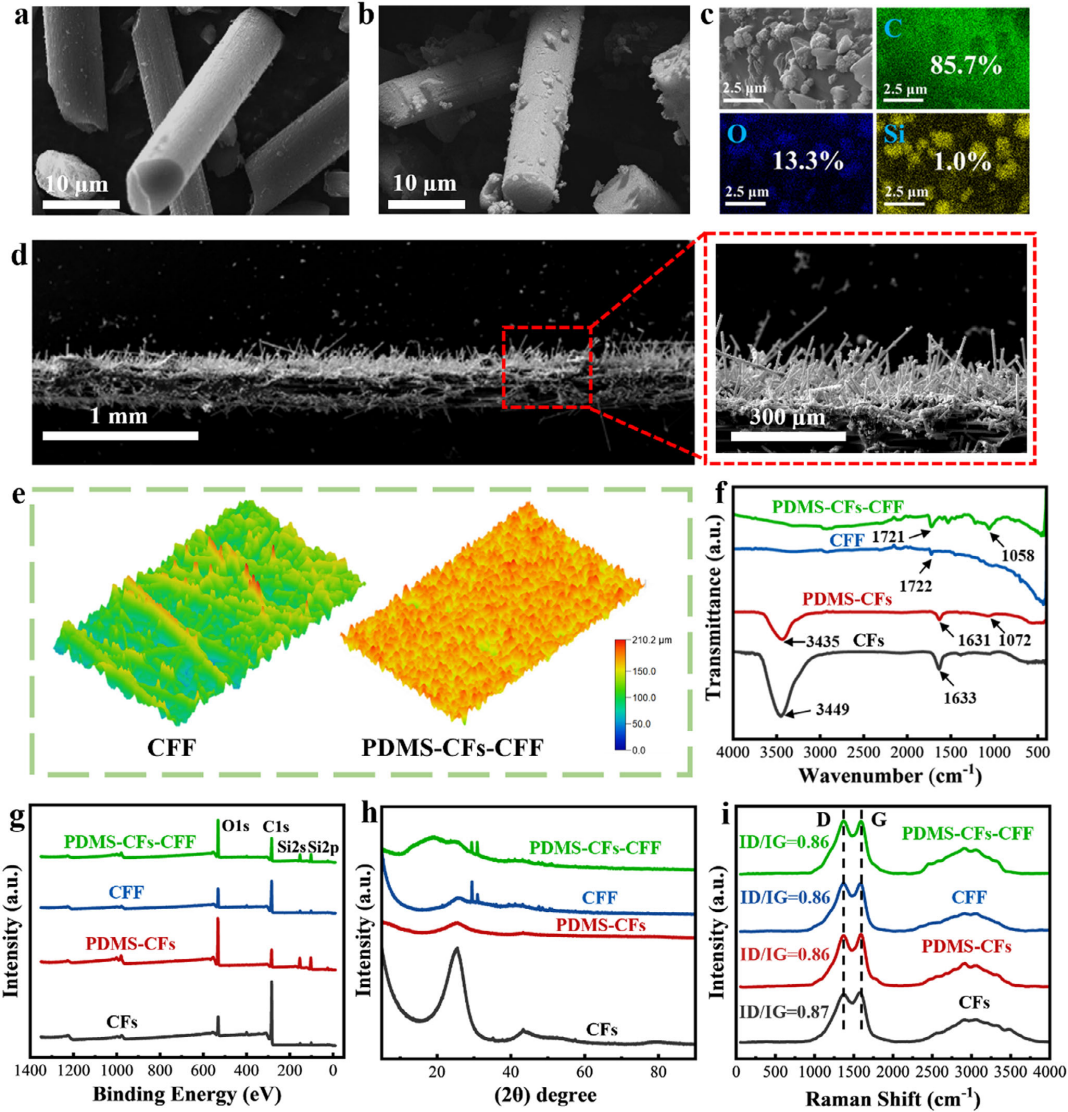
Characterization of surface morphology and composition. a,b) Scanning electron microscopy (SEM) images of CFs and PDMS‐CFs. c) EDS spectrum of PDMS‐CFs. d) SEM and high‐magnification SEM images of PDMS‐CFs‐CFF. e) 3D height profile images of CFF and PDMS‐CFs‐CFF surfaces. f) FTIR spectra of CFs, PDMS‐CFs, CFF, and PDMS‐CFs‐CFF. g) XPS spectra. h) XRD patterns. i) Raman spectra.

**Figure** **2**a,b display the microstructures of CFs and PDMS‐CFs, respectively. The surface of untreated CFs is smooth, whereas after high‐temperature heat treatment, PDMS is uniformly distributed as particles on the CF surface. Additionally, energy‐dispersive spectroscopy (EDS) characterization of PDMS‐CFs surface elements (Figure 2c) reveals the presence of C, Si, and O, with respective contents of 85.7%, 1%, and 13.3%. Beyond the intrinsic C element in CFs, the detection of Si and O confirms the successful modification of PDMS nanoparticles on the CF surface. On the other hand, gaps exist between individual carbon fibers in CFF, facilitating vapor escape. However, the CFF surface is flat (Figure S1, Supporting Information), which is detrimental to light absorption. To address this, we employed an electrostatic flocking process to integrate PDMS‐CFs onto the CFF surface, forming a special 3D structure (Figure 2d) that promotes multiple light reflections and enhances light absorption. Consistent with PDMS‐CFs, the PDMS‐CFs‐CFF surface also contains C, Si, and O elements (Figure S2, Supporting Information), indicating that the electrostatic flocking process preserved the PDMS‐CFs structure. Given that surface roughness is critical for efficient light absorption and superhydrophobic performance, the material surface morphology was characterized using a 3D topography profiler and confocal microscopy (Figure 2e; Figure S3, Supporting Information, respectively). As shown, the CFF surface is smooth, while the PDMS‐CFs‐CFF surface is irregular, confirming that the electrostatic flocking process successfully constructed a rough, special 3D structure on the PDMS‐CFs‐CFF surface.

Figure 2f presents the fourier‐transform infrared spectroscopy (FTIR) spectra of CFs, PDMS‐CFs, CFF, and PDMS‐CFs‐CFF. In the CFs spectrum, the peak at 3449 cm^−1^ corresponds to the stretching vibration of ‐OH, while the peak at 1633 cm^−1^ is attributed to the stretching vibration of C = C. In the PDMS‐CFs spectrum, the ─OH peak at 3435 cm^−1^ is significantly attenuated, which arises from the removal of moisture of CFs during thermal treatment. A new peak at 1072 cm^−1^, corresponding to the stretching vibration of the Si─O─Si bond, emerges, indicating the successful modification of PDMS nanoparticles onto the CFs surface after thermal treatment. Additionally, a Si─O─Si bond stretching vibration peak (1058 cm^−1^) is detected in the PDMS‐CFs‐CFF spectrum, confirming that PDMS‐CFs were successfully integrated onto CFF via the electrostatic flocking process. To further investigate the elemental composition and chemical bond states of PDMS‐CFs‐CFF, X‐ray photoelectron spectroscopy (XPS) was conducted, with results shown in Figure 2g. As depicted, only the characteristic peaks of C1s (284.1 eV) and O1s (532.3 eV) are detected in CFs and CFF, whereas new characteristic peaks corresponding to Si 2p (102.2 eV) and Si 2s (154.4 eV) emerge in PDMS‐CFs and PDMS‐CFs‐CFF. This confirms that PDMS‐CFs were successfully integrated into CFF while retaining their original structure. Furthermore, X‐ray diffraction (XRD) patterns illustrate the crystal structures of CFs, PDMS‐CFs, CFF, and PDMS‐CFs‐CFF (Figure 2h). CFs exhibit a sharp, intense diffraction peak ≈2θ = 26°, corresponding to the (002) crystal plane, indicative of an amorphous carbon structure. The diffraction peak near 2θ = 26° in PDMS‐CFs is significantly weakened, as graphite microcrystalline edges of CFs preferentially oxidize at high temperatures, leading to defects and microcracks. Raman spectroscopy (Figure 2i) further confirms that the ID/IG ratio of PDMS‐CFs‐CFF is lower than that of CFs, indicating a reduction in the average size of sp^3^ domains, consistent with the XRD results.

### 2.2 Photothermal Performance and Water Management of PDMS‐CFs‐CFF‐SF

**Figure 3:**
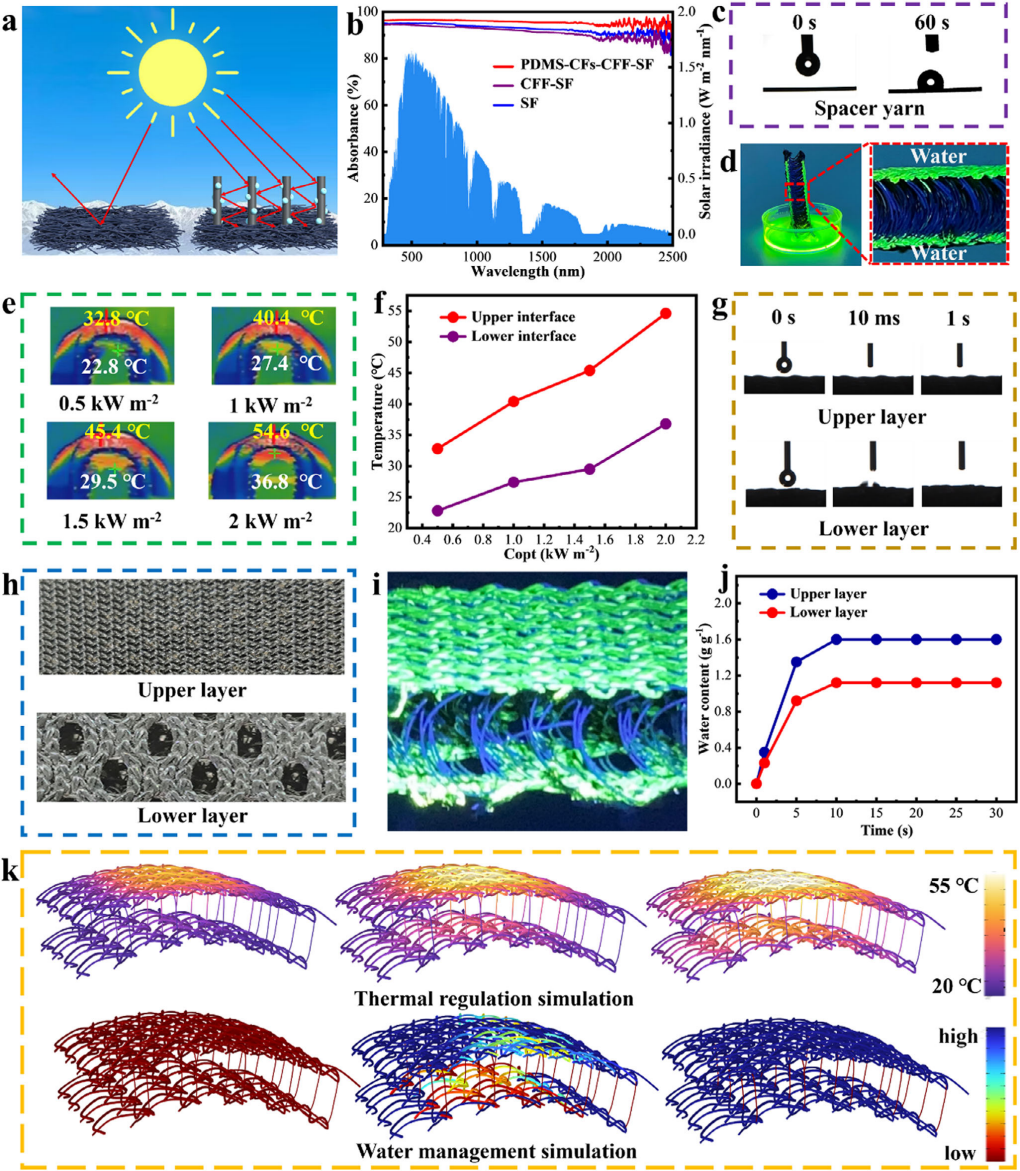
Thermal management and water management characterization of PDMS‐CFs‐CFF‐SF. a) Schematic diagram illustrating the reflection process of sunlight on different samples. b) Absorption spectra of PDMS‐CFs‐CFF‐SF, CFF‐SF, and SF in the UV‐vis‐NIR (300–2500 nm), along with the standard solar radiation spectrum. c) Water contact angle of the spacer yarns. d) Optical image of wet SF under ultraviolet light illumination. e) Infrared thermal images of PDMS‐CFs‐CFF‐SF in the wet state under different solar intensities. f) Stabilized temperatures of the upper and lower interfaces of wet PDMS‐CFs‐CFF‐SF under different solar intensities. g) Water contact angles of the upper and lower layers of SF. h) Optical images of the upper and lower layers of SF. i) Water distribution image of wet SF. j) Water content of the upper and lower layers of SF at different time points. k) COMSOL simulation of self‐regulating heat distribution and water supply matching in PDMS‐CFs‐CFF‐SF.

The surface of PDMS‐CFs‐CFF‐SF forms a special 3D structure with PDMS‐CFs via electrostatic flocking, which enables multiple reflections of incident light and is beneficial for enhancing the light absorption efficiency of the solar evaporator (**Figure** **3**a). Based on the aforementioned design, the light absorption characteristics of different samples in the dry state were tested using a ultraviolet‐visible‐near‐infrared‐region (UV–vis–NIR) spectrometer (Figure 3b). As depicted, PDMS‐CFs‐CFF‐SF exhibits an absorption rate of ≈95.6% across the UV–vis–NIR region (300–2500 nm), higher than that of CFF‐SF (≈90.37%) and SF (≈92.1%). This high absorbance is attributed to the synergistic effect of its special 3D structure and the broadband light absorption of CFs. In contrast, CFF‐SF shows lower absorbance than SF due to the flat CFF surface, which causes incident light reflection—conclusions supported by reflectance test results (Figure S4, Supporting Information). The photothermal conversion performance under varying light intensities was investigated by recording temperature changes of wet PDMS‐CFs‐CFF‐SF over time. As shown in Figure S5 (Supporting Information), PDMS‐CFs‐CFF‐SF heats up rapidly under different light intensities, reaching 38.6 °C in just 60 s under 1 kW m^−2^ illumination—indicating efficient conversion of solar energy into thermal energy. COMSOL Multiphysics simulations of its heating process showed good agreement with experimental data (Figure S5, Supporting Information), further confirming its excellent photothermal conversion performance. Given that the hydrophobicity of spacer yarns is critical to thermal regulation in PDMS‐CFs‐CFF‐SF, their hydrophobicity was characterized via water contact angle measurements. As shown in Figure 3c, the spacer yarns exhibit a contact angle of 111.3°, confirming hydrophobicity. Fluorescence testing was subsequently used to characterize the hydrophilic‐hydrophobic properties of different regions in the spacer fabrics (Figure 3d): the upper and lower layers of SF are filled with green fluorescence (indicating complete water immersion), while the spacer yarn layer shows no green fluorescence (indicating no water immersion), thus verifying the spacer yarns’ hydrophobic nature. The unique weaving process and material selection of SF ensure the independence of its upper and lower layers—water in the upper layer does not diffuse to the lower layer via spacer yarns. Under low solar intensity, heat accumulates at the upper interface of PDMS‐CFs‐CFF‐SF for localized heating, while the lower interface, without heat transfer from the upper layer, absorbs environmental energy to facilitate cold evaporation. Conversely, under high solar intensity, when excess heat accumulates at the upper interface, spacer yarns can directionally transfer this surplus heat to the lower interface, enabling dual‐interface evaporation. Additionally, infrared thermal images and stable temperatures of the upper and lower interfaces of wet PDMS‐CFs‐CFF‐SF under different light conditions are recorded in Figure 3e,f, respectively. As light intensity increases, the lower interface temperature of the PDMS‐CFs‐CFF‐SF system rises continuously—demonstrating that excess heat from the upper interface can be directed to the lower interface via spacer yarns, enabling automatic thermal regulation between the two interfaces and maximizing heat utilization for vapor generation.

Building on the aforementioned demonstration of the adaptive thermal regulation properties of PDMS‐CFs‐CFF‐SF, further investigations were conducted on its water management performance to validate its water‐thermal balance characteristics. As shown in Figure 3g, both the upper and lower layers of SF can fully absorb droplets within 1 s, indicating effective water transport within the evaporator's upper and lower interfaces. As noted earlier, since the heat acquired at the lower interface of PDMS‐CFs‐CFF‐SF primarily stems from the directed transfer of excess heat from the upper interface, a heat differential exists between the two interfaces. To address this, SF in this study employs a specialized surface structure with “small pores in the upper layer” and “large pores in the lower layer” (Figure 3h) to regulate water supply between the upper and lower interfaces, aiming to match their heat distribution. Fluorescence testing was used to observe water distribution (Figure 3i): the upper layer turned completely green, while the lower layer displayed black, large pores, demonstrating that the upper layer contains more water than the lower layer. Using the water content calculation Equation (1), the water content of the upper and lower layers at different time points was further quantitatively characterized (Figure 3j). As depicted, both layers reached water saturation within 10 s, with the upper layer exhibiting a water content of 1.6 g g^−1^, significantly higher than the lower layer's 1.12 g g^−1^.

Building on the aforementioned experiments, COMSOL Multiphysics was employed to simulate the heat and water distribution within the PDMS‐CFs‐CFF‐SF system, aiming to validate its self‐regulating heat management and water supply adaptation characteristics from a numerical simulation perspective. As illustrated in Figure 3k, under low solar intensity, heat accumulates and localizes at the upper interface of the PDMS‐CFs‐CFF‐SF system, while the lower interface absorbs environmental energy to facilitate cold evaporation. With increasing solar intensity, excess heat emerges at the upper interface, and spacer yarns directionally transfer this surplus heat to the lower interface, enabling full heat utilization. Additionally, water management simulations reveal that at the initial stage, both the upper and lower interfaces of the sample are dry. Upon water supply, the upper interface, benefiting from its “small‐pore structure,” achieves rapid water replenishment, resulting in higher water content. In contrast, the lower interface, with its large‐pore structure, contains less water, which matches the heat it receives. Overall, COMSOL simulation results are in good agreement with experimental observations, confirming the evaporator's self‐regulating heat properties and water supply adaptation characteristics.

### 2.3 Durability of PDMS‐CFs‐CFF‐SF

**Figure 4:**
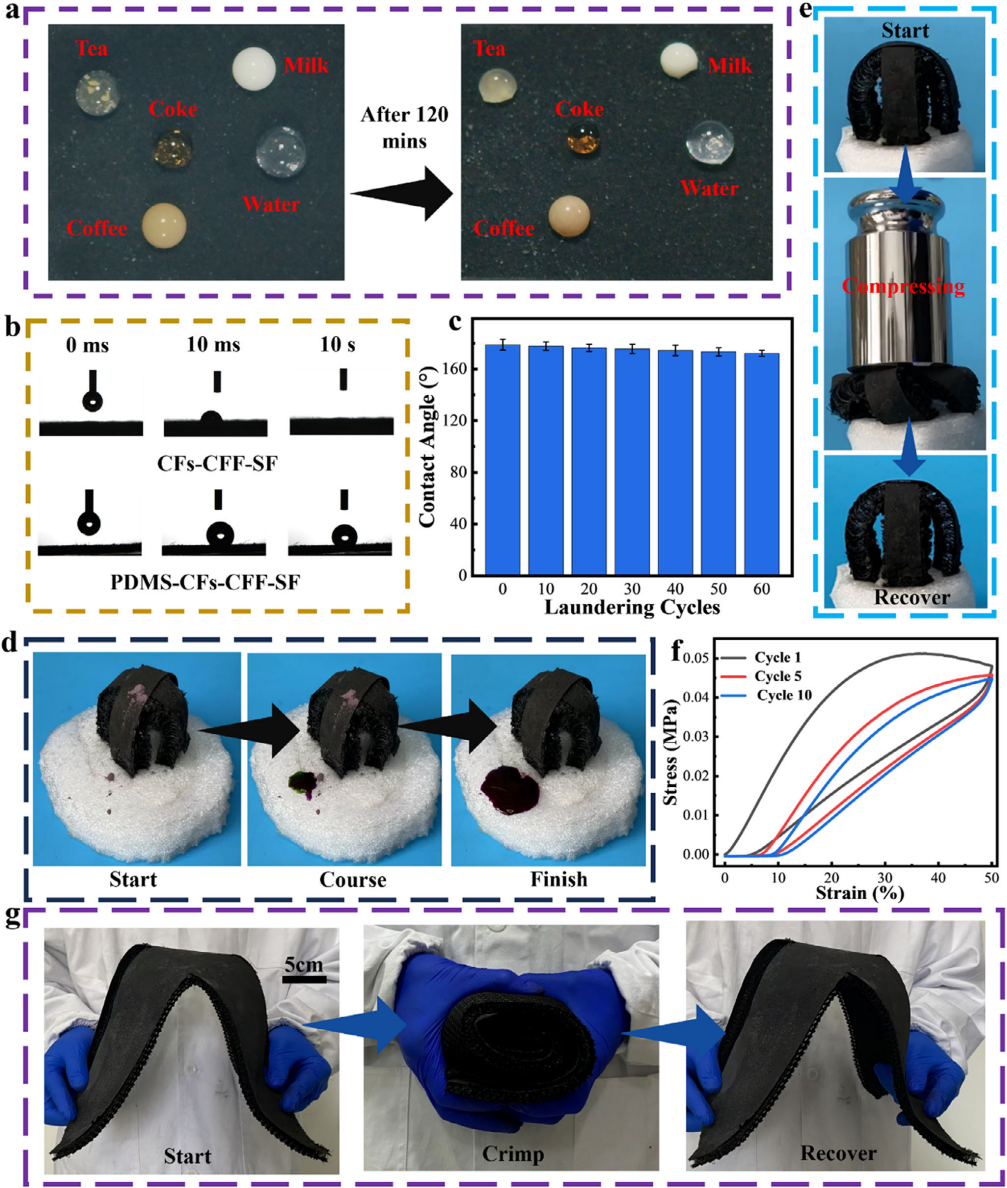
Antifouling performance and mechanical properties of PDMS‐CFs‐CFF‐SF. a) Optical images of tea, milk, cola, water, and coffee on the surface of PDMS‐CFs‐CFF‐SF. b) Water contact angle images of CFs‐CFF‐SF and PDMS‐CFs‐CFF‐SF. c) Water contact angles of PDMS‐CFs‐CFF‐SF after different washing cycles. d) Self‐cleaning process of PDMS‐CFs‐CFF‐SF. e) Compression recovery images of PDMS‐CFs‐CFF‐SF. f) Stress‐strain curves of SF after 10 cycles at 50% strain. g) Curling recovery images of large‐sized samples.

Solar evaporators deployed outdoors for extended periods may accumulate surface dust and impurities, which can impair the light absorption of photothermal materials and consequently reduce evaporation efficiency. Thus, solar evaporators should possess excellent anti‐soiling and self‐cleaning capabilities. As shown in **Figure** **4**a, PDMS‐CFs‐CFF‐SF exhibits pronounced hydrophobicity toward various liquids, including water, coffee, milk, cola, and tea—attributed to its ultra‐high water contact angle (178.8 ± 2.6°, Figure 4b). Given that solar evaporators operate in aqueous environments over long durations, their washing resistance is also critical for practical application. Washing tests were conducted to evaluate the washing resistance performance of PDMS‐CFs‐CFF‐SF (Figure 4c). As the number of washes increased, its water contact angle remained largely unchanged; even after 60 washes, the contact angle remained at 172.0 ± 2.3°, demonstrating excellent water resistance. To assess self‐cleaning performance, Rhodamine B (RB) dye powder was placed on the surfaces of PDMS‐CFs‐CFF‐SF, CFs‐CFF‐SF, and SF, followed by rinsing with a fluorescent agent‐containing solution. As observed, CFs‐CFF‐SF and SF lacked self‐cleaning ability, with most RB remaining adhered to their surfaces. The absorbed fluorescent solution caused their surfaces to appear red under ultraviolet light (Figure S6, Supporting Information). In contrast, RB on PDMS‐CFs‐CFF‐SF was completely removed, and the fluorescent solution slid off without leaving traces (Figure 4d; Movie S1, Supporting Information), confirming the excellent self‐cleaning properties of PDMS‐CFs‐CFF‐SF.

Excellent mechanical properties are also critical for ensuring the long‐term reliability of solar evaporators. When a 5 g weight is placed on the surface of PDMS‐CFs‐CFF‐SF, it remains stable with minimal deformation (Figure S7, Supporting Information), demonstrating sufficient mechanical strength. Under the pressure of a 500 g weight, PDMS‐CFs‐CFF‐SF deforms, but it can self‐recover to its initial state after the weight is removed, indicating excellent recovery capability (Figure 4e). Additionally, 10 cyclic compression tests were conducted on SF at 50% strain (Figure 4f). As observed, no significant plastic deformation occurred after 10 cycles, further confirming that PDMS‐CFs‐CFF‐SF possesses excellent flexibility and elastic recovery. More importantly, PDMS‐CFs‐CFF‐SF can be fabricated in large sizes (Figure S8, Supporting Information), with a cost of only 13.2 $ m^−2^ (Table S1, Supporting Information). In addition, the large‐sized PDMS‐CFs‐CFF‐SF is foldable (Figure S9, Supporting Information) and rollable (Figure 4g), which lays a foundation for convenient storage and transportation in large‐scale applications.

### 2.4 Evaporation Performance of PDMS‐CFs‐CFF‐SF

**Figure 5:**
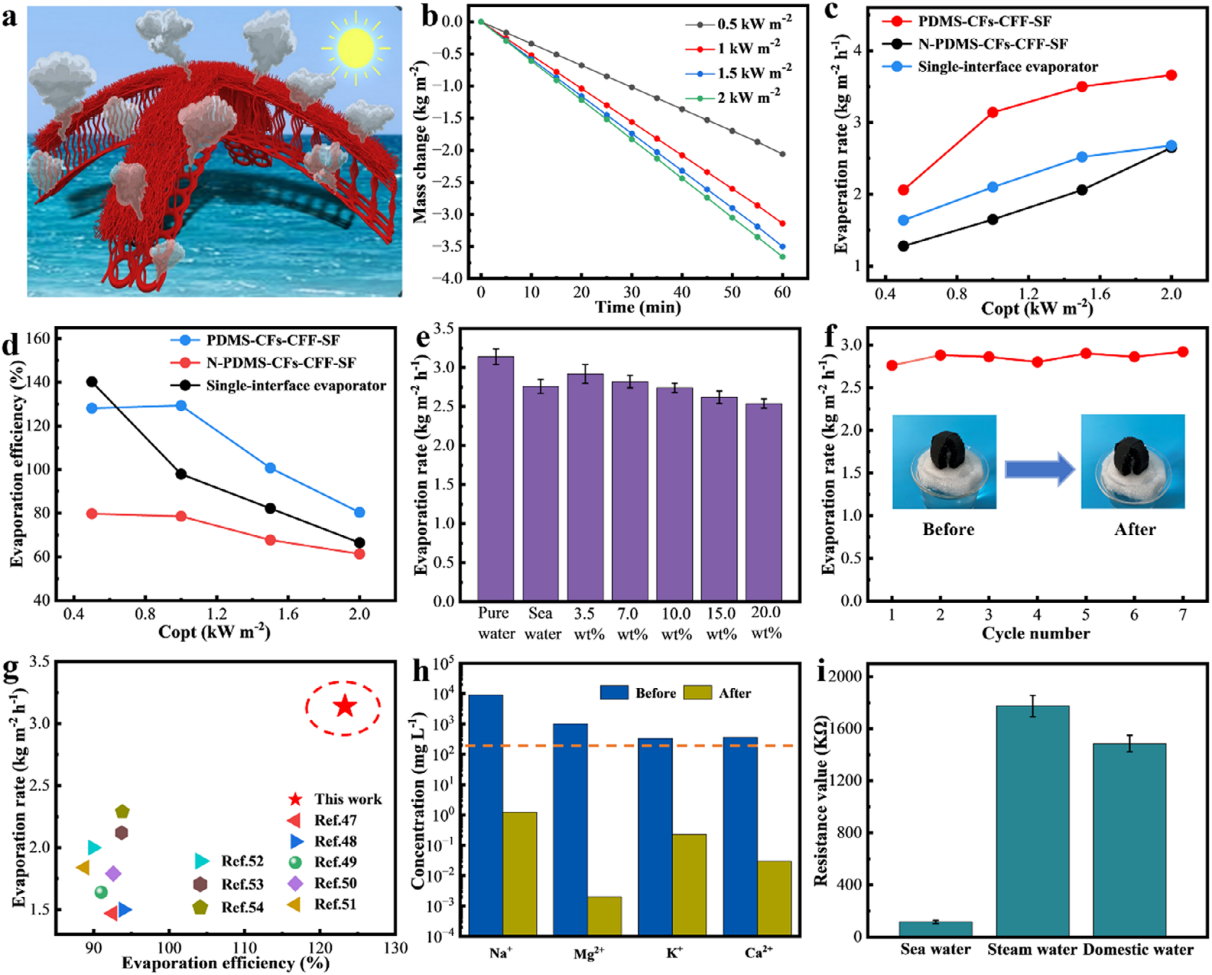
Water evaporation performance of PDMS‐CFs‐CFF‐SF. a) Schematic diagram illustrating the evaporation mechanism of PDMS‐CFs‐CFF‐SF. b) Mass change over time of PDMS‐CFs‐CFF‐SF under different solar intensities. c) Curves of evaporation rate changes for PDMS‐CFs‐CFF‐SF, N‐PDMS‐CFs‐CFF‐SF, and single‐interface evaporator under different solar intensities. d) Curve of evaporation efficiency variation. e) Diagram of evaporation rates of PDMS‐CFs‐CFF‐SF in freshwater, seawater, and brines with different salt concentrations. f) Evaporation efficiency and optical image of PDMS‐CFs‐CFF‐SF after 7 h of continuous desalination in simulated seawater. g) Comparison of evaporation performance between PDMS‐CFs‐CFF‐SF and previously reported CFs‐based ISSGs. h) Concentrations of four major ions before and after seawater desalination (dashed lines represent WHO‐established safe salinity standards). i) Resistivity images of Bohai Sea seawater, collected steam water, and domestic water.

As shown in **Figure** **5**a, 3D knitting technology and octopus‐inspired structure design endow PDMS‐CFs‐CFF‐SF with a large evaporation area and multiple steam escape pathways. Additionally, the air layer formed between its bottom and the water surface provides excellent thermal insulation. To visually characterize the solar‐driven water evaporation performance of PDMS‐CFs‐CFF‐SF, this study recorded water mass changes over time under different solar radiation conditions. As depicted in Figure 5b, the mass losses of PDMS‐CFs‐CFF‐SF after 1 h of irradiation at light intensities of 0.5, 1, 1.5, and 2 kW m^−2^ were 2.06, 3.14, 3.5, and 3.66 kg m^−2^, respectively. Notably, mass loss increases with rising light intensity, demonstrating the excellent photothermal sensitivity of PDMS‐CFs‐CFF‐SF. For comparison, the evaporation rates of PDMS‐CFs‐CFF‐SF, N‐PDMS‐CFs‐CFF‐SF, and the single‐interface evaporator under different solar irradiances are presented in Figure 5c. Thanks to its excellent structural design, the evaporation rate of PDMS‐CFs‐CFF‐SF was much higher than that of N‐PDMS‐CFs‐CFF‐SF and the single‐interface evaporator across all tested solar irradiances. In addition, the evaporation efficiencies of the three evaporators were calculated via Equations (2) and (3), with their comparisons shown in Figure 5d. Under 0.5 kW m^−2^ irradiation, the evaporation efficiency of PDMS‐CFs‐CFF‐SF was comparable to that of the single‐interface evaporator but significantly higher than that of N‐PDMS‐CFs‐CFF‐SF. Under 1, 1.5, and 2 kW m^−2^ irradiation, the evaporation efficiency of PDMS‐CFs‐CFF‐SF was notably higher than those of the other two evaporators, further demonstrating that the adaptive heat regulation of PDMS‐CFs‐CFF‐SF enables more heat to be utilized for water evaporation.

Additionally, the saltwater evaporation performance of PDMS‐CFs‐CFF‐SF was investigated to validate its desalination capability in practical applications. As shown in Figure 5e, for seawater collected from the Bohai Sea (Figure S10, Supporting Information), the evaporation rate of PDMS‐CFs‐CFF‐SF reached 2.76 kg m^−2^ h^−1^, demonstrating its potential for practical use. Additionally, the evaporation rates of PDMS‐CFs‐CFF‐SF were tested across different salt concentrations (3.5–20 wt.%), yielding values of 2.92, 2.82, 2.74, 2.62, and 2.54 kg m^−2^ h^−1^, respectively. As salt concentration increased, the evaporation rate of PDMS‐CFs‐CFF‐SF decreased slightly, which can be attributed to the reduced saturated vapor pressure induced by the osmotic effect of high‐salinity water. Nevertheless, PDMS‐CFs‐CFF‐SF still demonstrated effective handling of high‐concentration saltwater. To further assess desalination performance, its evaporation rate was measured after prolonged operation at 1 kW m^−2^ (Figure 5f). After 7 h of evaporation in 3.5 wt.% simulated saltwater, no salt crystallization was observed on the surface of PDMS‐CFs‐CFF‐SF (owing to the differential structure design of the upper surface with a hydrophilic upper layer and a hydrophobic lower layer, which effectively prevented the transmission of high‐concentration salt water to the evaporator surface), and its evaporation performance remained stable, confirming excellent continuous operational capability. Compared to typical CFs‐based ISSGs reported in previous literature, the PDMS‐CFs‐CFF‐SF developed in this study exhibits advantages in both evaporation rate and efficiency (Figure 5g).^[^ ^47^, ^48^, ^49^, ^50^, ^51^, ^52^, ^53^, ^54^ ^]^

Further, a laboratory‐fabricated sealing device was used to collect the generated steam condensate, and ion concentrations in the condensate were analyzed via inductively coupled plasma optical emission spectrometer (ICP‐OES) (Figure 5 h and Figure S11, Supporting Information). Results showed that the concentrations of Na⁺, Mg^2^⁺, K⁺, and Ca^2^⁺ in desalinated Bohai Sea water were 1.215, 0.002, 0.226, and 0.03 mg L^−1^, respectively, far below the safety limits specified by the World Health Organization (WHO). Additionally, water quality was further evaluated by measuring electrical resistance with a multimeter (Figure 5i). The resistance of the condensate collected after desalinating Bohai Sea water using PDMS‐CFs‐CFF‐SF increased from 115.56 KΩ to 1.7774 MΩ, which even exceeds the drinking water standard (1.486 MΩ). These results confirm that seawater desalinated via PDMS‐CFs‐CFF‐SF meets drinking water criteria. In summary, PDMS‐CFs‐CFF‐SF integrates efficient light absorption, favorable hydrothermal balance, a large evaporation area, multiple steam escape pathways, and excellent mechanical properties, ultimately achieving an exceptional evaporation rate and efficiency while maintaining durability.

### 2.5 Multi‐Scenario Practical Application Exploration of PDMS‐CFs‐CFF‐SF

**Figure 6:**
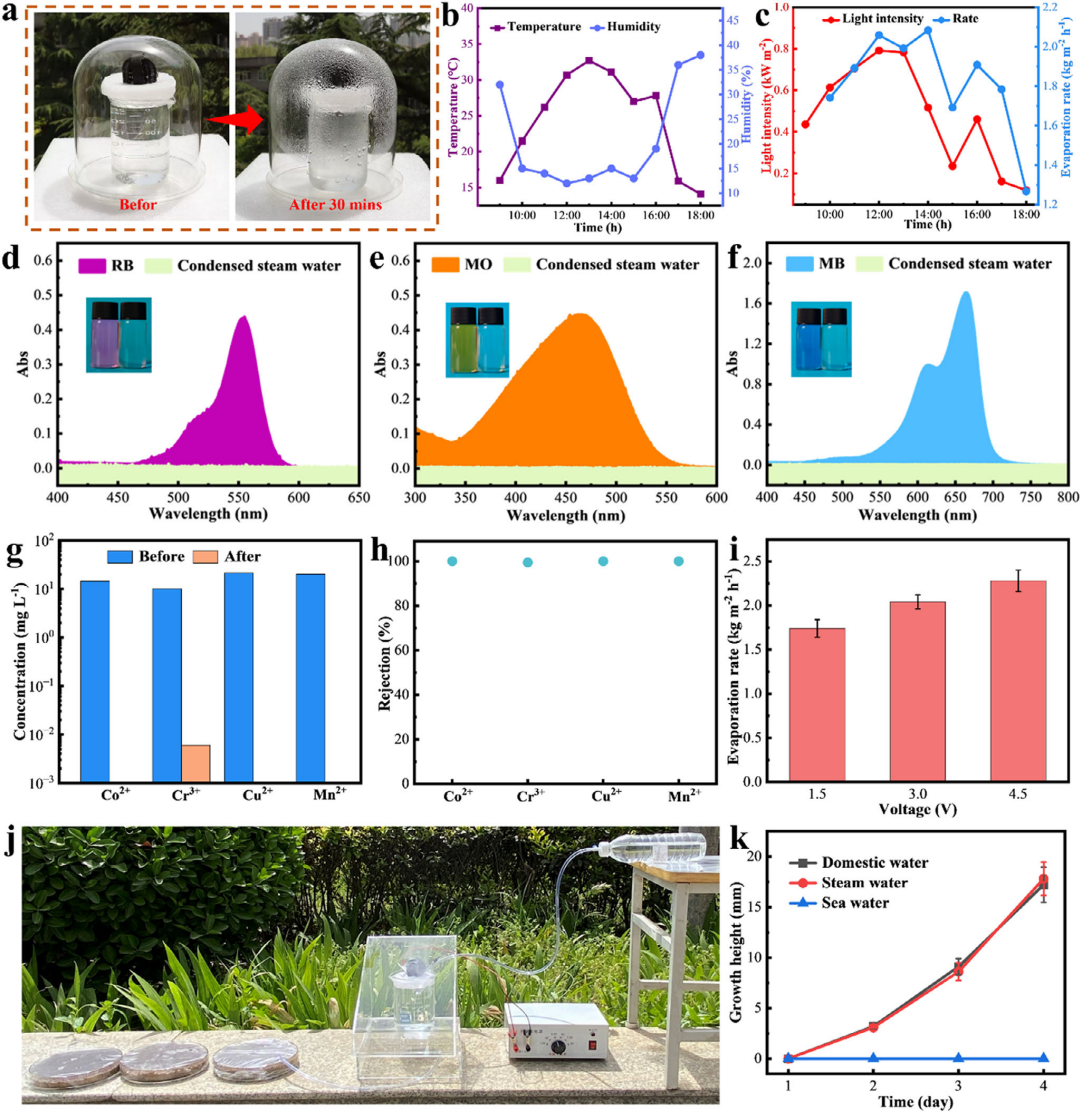
Outdoor experiments and wastewater purification. a) Optical images of the actual evaporation system, rapid steam generation, and water droplet condensation. b) Temperature and humidity during outdoor experiments. c) Solar intensity and evaporation rate. d–f) Absorption spectra of RB, MO, and MB solutions (10 mg L^−1^) before and after solar‐driven wastewater purification (insets in (d)–(f) show optical photographs of RB, MO, and MB before and after purification, respectively). g) Heavy metal ion concentrations before and after solar‐driven wastewater purification. h) Removal rates of heavy metal ions after solar‐driven wastewater purification. i) Evaporation rates of PDMS‐CFs‐CFF‐SF at different voltages. j) Optical images of the lettuce cultivation system. k) Time‐dependent growth height curves of lettuce seedlings irrigated with different water sources.

To further validate the capability of PDMS‐CFs‐CFF‐SF in treating real seawater under actual outdoor lighting conditions, a solar‐driven seawater desalination device was designed (**Figure** **6**a). The device comprises an outer chamber, an inner chamber, and a condensation cover: steam generated by PDMS‐CFs‐CFF‐SF condenses into water on the cover, flows down the outer chamber walls, and is collected at the bottom. Figure 6b,c show the environmental temperature, humidity, light intensity, and hourly evaporation rate recorded in Xi'an, China, from 9:00 to 18:00 on March 17, 2025. The evaporation rate exhibits a trend consistent with environmental temperature and light intensity. After 9 h of operation, the PDMS‐CFs‐CFF‐SF system collected up to 16.42 kg m^−2^ of clean water, with an average evaporation rate of 1.82 kg m^−2^ h^−1^ (Figure S12, Supporting Information). However, compared to laboratory conditions, outdoor experiment rates were lower. This is attributed to the significantly higher vapor pressure in the closed system than in open environments, water droplets and vapor adhering to the condensation cover scattering incident sunlight (reducing light intensity), and solar heating of the cover hindering continuous vapor condensation. Nevertheless, the results remain satisfactory, confirming that PDMS‐CFs‐CFF‐SF can efficiently process seawater under actual light conditions.

In addition to seawater desalination, the purification of dyeing and industrial wastewater represents another key application for solar evaporators. In this study, RB, methyl orange (MO), and methylene blue (MB) solutions were used to simulate chemical dye pollutants. The purification capacity of PDMS‐CFs‐CFF‐SF for dye‐containing wastewater was evaluated by analyzing the absorbance of solutions before and after treatment using a ultraviolet‐visible spectrophotometer, with results shown in Figure 6d–f. As indicated, the characteristic absorption peaks of RB, MO, and MB disappeared completely after purification, demonstrating ≈100 % dye removal. Subsequently, various heavy metal ions were used to simulate industrial wastewater, enabling assessment of PDMS‐CFs‐CFF‐SF's performance in solar‐driven purification of heavy metal‐contaminated industrial wastewater. The concentrations and removal rates of four metal ions (Co^2^⁺, Cr^3^⁺, Cu^2^⁺, and Mn^2^⁺) before and after purification are presented in Figure 6g,h, respectively. After treatment with PDMS‐CFs‐CFF‐SF, the concentration of Co^2^⁺ in simulated industrial wastewater was significantly reduced to 0.006 mg L^−1^, while Cr^3^⁺, Cu^2^⁺, and Mn^2^⁺ were undetectable, with removal rates exceeding 99%. Additionally, after purification using PDMS‐CFs‐CFF‐SF, the pH of dilute HCl and 0.2 mol L^−1^ NaOH solutions approached neutrality (Figure S13, Supporting Information), indicating its potential to balance pH under acidic or alkaline conditions. In summary, PDMS‐CFs‐CFF‐SF enables highly efficient solar‐driven wastewater purification.

Additionally, this work tested the vapor generation of PDMS‐CFs‐CFF‐SF under dark conditions using an external power supply to evaluate its all‐weather purification capability. The test setup is shown in Figure S14 (Supporting Information). Results indicate that at 1.5 V, the evaporation rate of PDMS‐CFs‐CFF‐SF reached 1.74 kg m^−2^ h^−1^, and it increased with rising voltage, reaching 2.28 kg m^−2^ h^−1^ at 4.5 V (Figure 6i). This confirms that PDMS‐CFs‐CFF‐SF can generate steam via Joule heating in the absence of light, enabling all‐weather stable purification. To further verify the practical feasibility of this technology, we designed a miniature desalination cultivation system comprising PDMS‐CFs‐CFF‐SF, a condensation unit, a power supply, and related cultivation equipment (Figure 6j). Steam generated by PDMS‐CFs‐CFF‐SF was condensed into freshwater via the condensation unit and directly used for lettuce cultivation. After four days of continuous operation, no salt crystals were observed on the surface of PDMS‐CFs‐CFF‐SF (Figure S15, Supporting Information), demonstrating excellent salt tolerance. Lettuce cultivated in this system showed growth rates comparable to those irrigated with tap water, both reaching ≈17 mm in height (Figure 6k; Figure S16, Supporting Information). In contrast, lettuce seeds irrigated with Bohai Sea seawater failed to germinate, highlighting the potential of this solar desalination system for vegetable cultivation.

**Figure 7:**
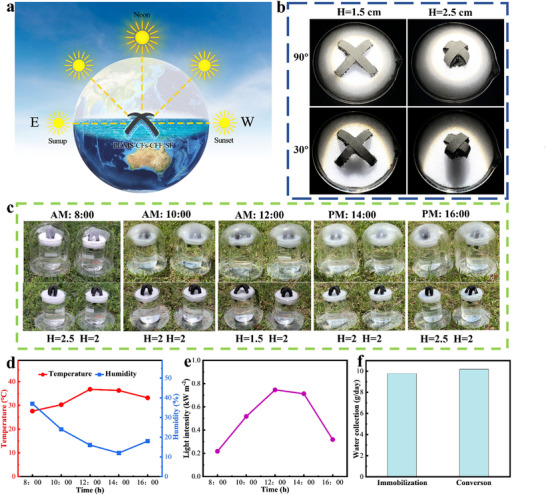
PDMS‐CFs‐CFF‐SF adapts to incident sunlight. a) Schematic diagram of the sun's movement trajectory. b) Projected area images of PDMS‐CFs‐CFF‐SF at different heights under xenon lamp illumination at various incident angles. c) Schematic diagram of the collection device and PDMS‐CFs‐CFF‐SF heights at different times. d) Temperature and humidity during outdoor experiments. e) Solar intensity. f) Water collection weight over one day for the control group (unchanged height) and the experimental group (changed height).

In practical conditions, the incident angle of sunlight changes constantly (**Figure** **7**a), and most existing solar evaporators cannot adapt to these angle variations, thus failing to fully utilize incident sunlight. Thanks to its octopus‐inspired structural design, PDMS‐CFs‐CFF‐SF can adapt to varying sunlight incident angles. First, by adjusting the angle of a xenon lamp to simulate different sunlight incident angles, the projected area of PDMS‐CFs‐CFF‐SF was used to characterize the actual light‐capturing area (Figure 7b). As shown, under vertical illumination, the projected area of the 1.5 cm‐high sample is larger than that of the 2.5 cm‐high sample; however, this relationship reverses at a 30° incident angle. Consequently, if PDMS‐CFs‐CFF‐SF is set to a higher height in the morning, adjusted to a lower height at noon, and then returned to a higher height in the evening, it can maintain a large light‐capturing area throughout the day. To further validate this hypothesis, an outdoor experiment was conducted where the sample height was continuously adjusted, and the process was recorded (Figure 7c). The temperature, humidity, and solar intensity during the experiment are shown in Figure 7d,e, respectively. After a full day of testing, the water collection volume of the control group (with unadjusted sample height) was significantly lower than that of the experimental group (with adjusted sample height) (Figure 7f). These results demonstrate that adjusting the height of PDMS‐CFs‐CFF‐SF according to the incident light angle can enhance the evaporator's evaporation rate.

## 3 Conclusion

Inspired by the natural structural features of marine octopuses, this study proposes a novel ISSG with an octopus‐inspired structure, denoted as PDMS‐CFs‐CFF‐SF. Fabricated via the integration of 3D knitting technology and electrostatic flocking processes, this ISSG features a unique biomimetic 3D architecture that provides additional vapor escape pathways and an expanded evaporation area, ensuring efficient vapor release and a larger light‐absorbing surface. Moreover, a cavity formed between the evaporator bottom and the water surface minimizes contact with bulk water, thereby reducing heat loss. The special 3D structure of PDMS‐CFs‐CFF‐SF enables efficient light harvesting, achieving a light absorption rate of ≈95.6% and ensuring effective solar‐to‐thermal conversion. More importantly, its specialized knitted coil‐wrapped structure allows self‐regulation of heat distribution between the upper and lower interfaces in response to variations in external light intensity, maximizing heat utilization for efficient water evaporation. Consequently, under 1 kW m^−2^ illumination, PDMS‐CFs‐CFF‐SF achieves a high evaporation rate of 3.14 kg m^−2^ h^−1^ and an evaporation efficiency of 129.32%. Notably, the height of PDMS‐CFs‐CFF‐SF is highly adjustable, enabling adaptation to sunlight at different incident angles; compared to a nonadjustable control group, it yields higher daily water production. Furthermore, PDMS‐CFs‐CFF‐SF exhibits remarkable purification performance for wastewater containing heavy metal ions or chemical dyes. Thanks to its excellent electrothermal properties, it achieves an evaporation rate of up to 1.74 kg m^−2^ h^−1^ under dark conditions with an input voltage of only 1.5 V, enabling efficient and stable all‐weather water purification. In summary, the adaptive dynamic hydrothermal balance achieved through this specialized biomimetic structure offers a new perspective for enhancing solar evaporator performance, aids in establishing energy matching for ISSG systems, and demonstrates significant potential for solar‐driven seawater desalination across diverse environmental conditions.

## 4 Experimental Section

### Materials

PDMS prepolymer (Sylgard 184A) and curing agent (Sylgard 184B) were obtained from Dow Corning Corporation. Carbon fibers (with a diameter of 7 µm and an aspect ratio ranging from 2:1 to 8:1) were supplied by Tanxi Technology (Shenzhen) Co., Ltd. Water‐based polyurethane was purchased from Tanshenxing Biotechnology Co., Ltd. Carbon fiber felt (20 g m^−2^) was acquired from Zhejiang Huayi New Materials Co., Ltd. All chemicals used were of analytical grade and required no further purification.

### Fabrication of SF

SF was fabricated using an E‐18 double‐needle‐bar Raschel knitting machine (GE296, Wuyang Textile Machinery Co., Ltd.) equipped with six guide bars (GB1–GB6). GB1 and GB2 weave the upper “small‐hole” layer of SF on the front needle bed with a chain‐link weft structure, while GB5 and GB6 form the lower “large‐hole” layer on the rear needle bed with a six‐hole mesh structure; GB3 and GB4 are responsible for knitting the spacer yarn layer, alternating between the front and rear needle beds to connect the two surface layers. The fabric has a thickness of 7 mm and a surface density of 680 g m^−^^2^, with 300 D/96 F polyester composite yarn used for the surface layer and 120 D polyester monofilament for the spacer yarns. Detailed weaving processes and structural parameters of SF are provided in Tables S2–S4 (Supporting Information). SF is black, suitable for rapid large‐scale commercial production, and exhibits excellent extensibility and flexibility (Figures S17 and S18, Supporting Information).

Fabrication of PDMS‐CFs‐CFF‐SF: PDMS (3 g) prepolymer and curing (0.3 g) agent were mixed thoroughly in a crucible. The mixture was then heated in an oven at 110 °C for 10 min to form a PDMS block. Next, CFs (0.1 g) were placed on the surface of the PDMS block, and the crucible was transferred to a muffle furnace, where it was heated to 350 °C at a rate of 5 °C min^−1^ and held at this temperature for 3 h to prepare PDMS‐CFs. The as‐prepared PDMS‐CFs were integrated onto CFF using an electrostatic flocking machine with an input voltage of 12 V to produce PDMS‐CFs‐CFF. Subsequently, SF was cut into a biomimetic X‐shape, and PDMS‐CFs‐CFF was sewn onto its upper layer, resulting in the successful preparation of high‐performance PDMS‐CFs‐CFF‐SF. For comparison, two types of control evaporators were prepared in this study: SF was cut into individual surface layers, onto which PDMS‐CFs‐CFF was sewn to obtain single‐interface evaporators; meanwhile, PDMS‐CFs‐CFF was directly sewn onto the surface of intact SF to fabricate N‐PDMS‐CFs‐CFF, which served as the other control group.

### Characterization

The microstructure of the samples was characterized using a scanning electron microscope (SEM, QUANTA‐450‐FEG, Shanghai Boyue Instrument Co., Ltd.) with an acceleration voltage of 20 kV. The elemental composition and content were analyzed via energy‐dispersive spectroscopy (EDS, Xplore 30). Surface roughness was evaluated using a 3D topography profiler (RTEC‐UP2000, USA) and a confocal microscope (OLS5100‐SAF, Japan). The chemical structure of the samples was analyzed using Fourier transform infrared spectroscopy (Shimadzu‐IRTracer 100, Shimadzu), X‐ray diffraction (XRD, XRD‐6100, Shimadzu), Raman spectroscopy (in Via, Renishaw), and X‐ray photoelectron spectroscopy (XPS, ESCALAB 250Xi, ThermoFisher Scientific). UV–vis–NIR spectrophotometry (Lambda 950, PerkinElmer) was used to determine light absorption properties. Hydrophilicity/hydrophobicity was characterized using a contact angle measuring instrument (OCA15EC, Dataphysics). The weight moisture content (mass of water per gram of dry sample) of different surface layers was calculated to assess water management performance at different time points, with the calculation Equation for weight moisture content *W*g as follows:

(1) $$W_{g}=\frac{M_{1}-M_{0}}{M_{0}}$$

In the Equation, *M*_0_ represents the weight of the surface layers in the dry state, and *M*_1_ denotes the weight of the surface layers in the wet state.

### Fluorescence Test

SF was cut into strips and immersed in a culture dish containing a fluorescent agent solution. After 30 min, the SF strips were irradiated with ultraviolet light to observe the distribution of the fluorescent agent.

### Self‐Cleaning Experiment of PDMS‐CFs‐CFF‐SF

RB powder was placed on the sample surface, followed by rinsing with a green fluorescent agent solution. The residues of RB and the fluorescent agent solution on the sample surface were then observed.

Mechanical property testing: Cyclic compression tests were performed using a UTM5504 electronic universal testing machine in accordance with Chinese standard GB/T 33614‐2017.

### Solar‐Driven Water Evaporation and Seawater Desalination Experiments of PDMS‐CFs‐CFF‐SF

The experimental environment was maintained at ≈25 °C and 50% humidity. Prepared samples were placed in beakers containing bulk water. A xenon lamp (Qx sun‐500, Jilin Qingxuan Technology Co., Ltd.) equipped with an AM 1.5 filter served as the simulated light source, with light intensity calibrated using a densitometer (TES‐1333, Taishi Electronic Industry Co., Ltd.). The temperature at the upper interface of the sample was recorded using a thermal infrared imaging camera (testo 875 Pro, Beijing Startech Co., Ltd.), while the lower interface temperature was measured with a thermocouple (DT1312, Shenzhen Temperature Measurement Instruments Electronic Tools Co., Ltd.). Mass changes of water during evaporation were recorded every 5 min using an electronic balance (YP6002, Shanghai Shuangxu Electronics Co., Ltd.) with a precision of 0.001 g. The evaporation rate was calculated using the following Equation:

(2) $$V=\frac{M}{ST}$$

where *M* is the mass loss of water during evaporation, *S* is the projected area of the sample, and *T* is the experimental evaporation duration. The Equation for calculating evaporation efficiency *𝜂* is as follows:

(3) $$\eta =\frac{V_{1\Delta }H_{1}}{Q_{i}}\times 100\%$$

Among these, *V*_1_ represents the evaporation rate induced by solar radiation (kg m^−2^ h^−1^), *∆H*_1_ denotes the enthalpy change associated with water evaporation, with specific calculation steps detailed in the supplementary materials, and *Q*_i_ indicates the incident solar irradiance intensity. In solar desalination experiments, PDMS‐CFs‐CFF‐SF was immersed in Bohai Sea water, and steam condensate was collected using a laboratory‐fabricated sealed device. The concentrations of Na⁺, Mg^2^⁺, K⁺, and Ca^2^⁺ in the condensate were determined via inductively coupled plasma optical emission spectrometry (ICP‐OES, Agilent 5800) to evaluate the seawater desalination capability of PDMS‐CFs‐CFF‐SF.

Salt resistance test of PDMS‐CFs‐CFF‐SF: For the salt resistance test, PDMS‐CFs‐CFF‐SF was placed in a beaker containing simulated seawater (3.5 wt.% NaCl solution) and subjected to continuous evaporation for 7 h under an illumination intensity of 1 kW m^−2^. The surface of PDMS‐CFs‐CFF‐SF was observed to detect the presence of NaCl crystals.

Solar‐driven wastewater purification experiments of PDMS‐CFs‐CFF‐SF: Solutions of 10 mg L^−1^ RB, MO, and MB were used to simulate chemical dye wastewater. The absorption spectra of these aqueous solutions were recorded using a UV–vis spectrophotometer (UV–vis, UV‐3600i Plus, Shimadzu) to evaluate the purification capacity of PDMS‐CFs‐CFF‐SF for dye‐containing wastewater. Simulated heavy metal wastewater containing Co^2^⁺, Cr^3^⁺, Cu^2^⁺, and Mn^2^⁺ was prepared using CoCl_2_ 6H_2_O, CrCl_3_ 6H_2_O, CuSO_4_, and MnSO_4_ H_2_O powders. PDMS‐CFs‐CFF‐SF was then placed in a beaker with this simulated heavy metal wastewater, and its purification capacity was analyzed by collecting the steam condensate. Additionally, dilute HCl and 0.2 mol L^−1^ NaOH solutions were used to simulate acidic and alkaline wastewater, respectively. The neutralization capacity of PDMS‐CFs‐CFF‐SF for such wastewater was evaluated by measuring the pH of the purified solution.

Finite element simulations were performed using COMSOL Multiphysics (Version 6.2) to model the thermal regulation and water transport behaviors of PDMS‐CFs‐CFF‐SF during the evaporation process, thereby verifying its advanced hydrothermal balance performance. The simulation setup was consistent with actual experimental conditions (ambient temperature: 25 °C; humidity: 50%). Due to the high computational load of kinetic simulations, modeling the entire sample would lead to computational challenges; thus, half of the sample area was selected for simulation. Notably, directed heat transfer and water transport are primarily achieved via spacer yarns and surface layers, while PDMS‐CFs‐CFF has a negligible impact on the final results of these processes. To simplify computational complexity and ensure result convergence, the upper interface was simplified to the surface layer when establishing the evaporator solid model. Consequently, the finite element model in this study consists of three main components: the upper surface layer, the spacer yarns layer, and the lower surface layer, with all yarns simplified as flexible cylinders. The solid heat transfer module in COMSOL was used to simulate the evaporator's thermal regulation process, with the top of the air domain set as a temperature boundary condition and the remaining boundaries defined as walls. Meanwhile, the rarefied fluid transport module was employed to model the evaporator's water management process. Mesh quality directly influences simulation accuracy: excessively dense meshes consume excessive computational resources, while overly coarse meshes fail to accurately capture internal details of the representative volume element model. Therefore, the maximum and minimum mesh element sizes were set to 1.92 and 0.24 mm, respectively. The finite element model and mesh partitioning are presented in Figures S19 and S20 (supplementary information), respectively.

## Conflict of Interest

The authors declare no conflict of interest.

## Author Contributions

N.N. and L.Y. contributed equally to this work. N.N. performed data curation, investigation, wrote the original draft, wrote, reviewed, and edited the final draft. L.Y. performed formal analysis, methodology, wrote the original draft, wrote, reviewed, and editedthe final draft. J.M. performed methodology, project administration, and supervision. W.F. performed resources, validation, and visualization. K.C. performed the investigation, methodology. W.H. performed an investigation, methodology. Y.W. performed project administration, software. Y.L. performed software, supervision. Y.L. performed project administration, resources. Z.S. performed supervision, validation. C.Z. performed conceptualization, funding acquisition, wrote the original draft, wrote, reviewed, and edited the final draft.

## Supporting information



Supporting Information

Supplemental Movie 1

## Data Availability

The data that support the findings of this study are available from the corresponding author upon reasonable request.

## References

[1] M. Shamsudduha , R. G. Taylor , M. I. Haq , S. Nowreen , A. Zahid , K. M. U. Ahmed , Science 2022, 377, 1315. 36108006 10.1126/science.abm4730

[2] Z. N. Garba , W. Zhou , I. Lawan , M. Zhang , Z. Yuan , Cellulose 2019, 26, 6241.

[3] M. Wang , B. L. Bodirsky , R. Rijneveld , F. Beier , M. P. Bak , M. Batool , B. Droppers , A. Popp , M. T. van Vliet , M. Strokal , Nat. Commun. 2024, 15, 880. 38321008 10.1038/s41467-024-44947-3 PMC10847517

[4] M. A. Shannon , P. W. Bohn , M. Elimelech , J. G. Georgiadis , B. J. Mariñas , A. M. Mayes , Nature 2008, 452, 301. 18354474 10.1038/nature06599

[5] J. D. Kocher , A. K. Menon , Energy Environ. Sci. 2023, 16, 4983.

[6] J. Guo , Y. Zheng , Z. Hu , C. Zheng , J. Mao , K. Du , M. Jaroniec , S. Z. Qiao , T. Ling , Nat. Energy. 2023, 8, 264.

[7] L. Saleh , T. Mezher , Renewable Sustainable Energy Rev. 2021, 150, 111465. 10.1016/j.rser.2021.111425 PMC8441177 34539218

[8] M. Hafiz , R. Alfahel , A. Altaee , A. H. Hawari , Sep. Purif. Technol. 2023, 309, 123007.

[9] Y. Yao , P. Zhang , F. Sun , W. Zhang , M. Li , G. Sha , L. Teng , X. Wang , M. Huo , R. M. DuChanois , T. Cao , Science 2024, 384, 333. 38669571 10.1126/science.adk0632

[10] W. Liu , J. L. Livingston , L. Wang , Z. Wang , M. del Cerro , S. A. Younssi , R. Epsztein , M. Elimelech , S. Lin , Nat. Rev. Methods Primers. 2024, 4, 10.

[11] H. Liu , B. Chen , Y. Chen , M. Zhou , F. Tian , Y. Li , J. Jiang , W. Zhai , Adv. Mater. 2023, 35, 2301596. 10.1002/adma.202301596 37037047

[12] H. Yu , H. Jin , M. Qiu , Y. Liang , P. Sun , C. Cheng , P. Wu , Y. Wang , X. Wu , D. Chu , M. Zheng , Adv. Mater. 2024, 36, 2414045. 10.1002/adma.202414045 39548925

[13] L. Qi , J. Meng , L. Yu , C. Zhi , J. Text. Res. 2025, 46, 122.

[14] P. Tao , G. Ni , C. Song , W. Shang , J. Wu , J. Zhu , G. Chen , T. Deng , Nat. Energy. 2018, 3, 1031.

[15] H. Xu , Y. Zhu , Z. Xu , M. Dasog , P. Wang , Cell Rep. Phy. Sci 2025, 6, 102313.

[16] S. W. Sharshir , A. W. Kandeal , A. Joseph , M. M. Elsayad , A. S. Abdullah , S. H. Jang , M. Elashmawy , G. B. Abdelaziz , N. M. Ghazaly , Z. Yuan , Appl. Therm. Eng. 2024, 254, 123869.

[17] C. Guo , E. Miao , J. Zhao , L. Liang , Q. Liu , Sol. Energy. 2019, 188, 1283.

[18] H. Ma , L. Yu , Z. Li , J. Chen , J. Meng , Q. Song , Y. Liu , Y. Wang , Q. Wu , M. Miao , C. Zhi , Small 2023, 19, 2304877. 10.1002/smll.202304877 37635127

[19] Y. Wu , J. Ma , S. Zang , W. Zhou , Z. Wang , M. Han , S. M. Osman , C. Wang , Y. Yamauchi , J. You , M. An , Chem. Eng. J. 2023, 472, 144600.

[20] B. Yang , Z. Zhang , P. Liu , X. Fu , J. Wang , Y. Cao , R. Tang , X. Du , W. Chen , S. Li , H. Yan , Nature 2023, 622, 499. 37704732 10.1038/s41586-023-06509-3

[21] S. Lin , H. Qi , P. Hou , K. Liu , J. Cleaner Prod. 2023, 391, 136148.

[22] L. Li , C. Xue , Q. Chang , X. Ren , N. Li , J. Yang , S. Hu , H. Xu , Adv. Mater. 2024, 36, 2401171. 10.1002/adma.202401171 38497304

[23] R. Niu , J. Ren , J. J. Koh , L. Chen , J. Gong , J. Qu , X. Xu , J. Azadmanjiri , J. Min , Adv. Energy Mater. 2023, 13, 2302451.

[24] D. Chu , Z. Tang , S. Hu , F. Yang , S. Qu , W. Mao , P. Yao , Chem. Eng. J. 2025, 515, 163724.

[25] Z. Fu , D. Zhong , S. Zhou , L. Zhang , W. Long , J. Z. , X. Wang , J. Xu , J. Qin , J. Gong , L. Li , Adv. Sci. 2024, 11, 2406474. 10.1002/advs.202406474 PMC11615747 39303161

[26] Y. Guo , C. M. Dundas , X. Zhou , K. P. Johnston , G. Yu , Adv. Mater. 2021, 33, 2102994. 10.1002/adma.202102994 34292641

[27] J. Sun , M. U. Farid , W. Xi , G. Lu , M. W. Boey , S. K. Ravi , P. H. L. Sit , A. K. An , Adv. Funct. Mater. 2025, 2416768.

[28] F. Zhao , Y. Guo , X. Zhou , W. Shi , G. Yu , Nat. Rev. Mater. 2020, 5, 388.

[29] J. Ma , X. Sun , L. Wang , M. An , M. Kim , Y. Yamauchi , N. Khaorapapong , Z. Yuan , Nano Energy 2025, 137, 110781.

[30] D. Deng , Q. Liang , C. Guo , C. Liu , ACS Sustain. Chem. Eng. 2024, 12, 1446.

[31] L. Li , L. Du , Y. Bai , Y. Wang , M. Hao , M. Liu , C. Ge , S. Wang , H. Zheng , L. Li , F. Sun , EcoMat 2023, 5, 12289.

[32] Z. Ma , L. Jiang , Y. Cao , Desalination 2024, 586, 117880.

[33] N. He , Y. Yang , H. Wang , F. Li , B. Jiang , D. Tang , L. Li , Adv. Mater. 2023, 35, 2300189. 10.1002/adma.202300189 36795916

[34] N. Xu , X. Hu , W. Xu , X. Li , L. Zhou , S. Zhu , J. Zhu , Adv. Mater. 2017, 29, 1606762. 10.1002/adma.201606762 28520092

[35] N. Yu , H. Hu , W. Xia , Z. Zhao , H. Cheng , J. Colloid Interface Sci. 2024, 658, 238. 38104406 10.1016/j.jcis.2023.12.059

[36] X. Yuan , J. Jiang , Z. Zhou , H. Pan , C. Wang , C. Y. Tay , J. Hu , L. Liu , B. Li , Y. Cai , M. Liu , Chem. Eng. J. 2024, 481, 148178.

[37] D. Yue , B. Li , D. Sun , H. Zhang , M. Liu , J. Yu , Nano Res. 2023, 16, 10358.

[38] S. Zhang , M. Li , C. Jiang , D. Zhu , Z. Zhang , Adv. Sci. 2024, 11, 2308665. 10.1002/advs.202308665 PMC11077647 38342614

[39] C. Chen , L. Xiong , X. Zhang , K. Tian , Z. Dai , Q. Fu , H. Deng , Mater. Horiz. 2023, 10, 5161. 37712534 10.1039/d3mh01105d

[40] C. Wang , K. Xu , G. Shi , D. Wei , Adv. Energy Mater. 2023, 13, 2300134.

[41] Y. Yang , D. Wang , W. Liao , H. Zeng , Y. Wu , L. Li , W. Feng , J. Xue , H. Cao , J. Chen , Y. Huang , Adv. Fiber Mater. 2024, 6, 1026.

[42] S. Wu , G. Xiong , H. Yang , B. Gong , Y. Tian , C. Xu , Y. Wang , T. Fisher , J. Yan , K. Cen , T. Luo , Adv. Energy Mater. 2019, 9, 1901286.

[43] T. Zhang , Y. Lv , P. Cao , Z. Song , Q. Wang , Y. Guo , L. Hong , Y. Chen , Chem. Eng. J. 2025, 515, 163828.

[44] A. Bruno , M. Martucci , F. S. Cafagna , R. Sparvoli , O. Adriani , G. C. Barbarino , G. A. Bazilevskaya , R. Bellotti , M. Boezio , E. A. Bogomolov , M. Bongi , Astrophys. J. Lett. 2021, 917, L21.

[45] D. Wei , C. Wang , G. Shi , J. Zhang , F. Wang , P. Tan , Z. Zhao , Y. Xie , Adv. Mater. 2024, 36, 2309507. 10.1002/adma.202309507 38273713

[46] X. Xiao , Y. Wang , L. Ren , L. Lin , M. Wang , Y. Wang , D. Liu , X. Liu , Q. Zhang , W. Xu , Sep. Purif. Technol. 2024, 336, 126329.

[47] T. Li , Q. Fang , X. Xi , Y. Chen , F. Liu , J. Mater. Chem. A. 2019, 7, 586.

[48] T. Jiang , C. Guo , S. Qian , J. Wang , K. Zhu , H. Xue , J. Tian , Sep. Purif. Technol. 2025, 371, 133378.

[49] M. Zou , Y. Zhang , Z. Cai , C. Li , Z. Sun , C. Yu , Z. Dong , L. Wu , Y. Song , Adv. Mater. 2021, 33, 2102443. 10.1002/adma.202102443 34288134

[50] W. Chong , R. Meng , Z. Liu , Q. Liu , J. Hu , B. Zhu , D. K. Macharia , Z. Chen , L. Zhang , Adv. Fiber Mater. 2023, 5, 1063.

[51] D. Xu , C. Ge , Z. Chen , Y. Liu , H. Du , H. Gong , C. Gao , Z. Shen , W. Xu , K. Liu , ACS Appl. Mater. Interfaces. 2022, 14, 52087. 36376264 10.1021/acsami.2c18300

[52] L. Yang , M. Sun , G. Huang , Y. Zhan , Y. Gao , X. Zhou , B. Sun , L. Lyu , Chem. Eng. J. 2024, 495, 153180.

[53] X. Xiao , L. Pan , T. Chen , M. Wang , L. Ren , B. Chen , Y. Wang , Q. Zhang , W. Xu , Engineering 2023, 30, 153.

[54] L. Jin , L. Zhang , H. Liang , Y. Ao , S. Wang , D. Sun , Chem. Eng. J. 2024, 497, 154469.

