# Supplementary material for: Biomimetic Adaptive Hydrothermal Balance Fabric‐Based Dual‐Interface Solar Evaporator for Efficient and Stable Desalination
Source: Adv Sci (Weinh). 2025 Dec 12;13(10):e21275. doi: 10.1002/advs.202521275 (PMC12915201; doi:10.1002/advs.202521275)
Supplement: Supplementary file 1 — Supporting Information [file ADVS-13-e21275-s002.docx]

Supporting Information for

**Biomimetic Adaptive Hydrothermal Balance Fabric-Based Dual-Interface Solar Evaporator for Efficient and Stable Desalination**

Ning Niu ^a, b^, Lingjie Yu ^a, b^, Jiaguang Meng ^a, b^, Wei Fan ^a, b^, Kaili Chen ^a, b^, Wanwan He ^a, b^, Yongzhen Wang ^a, b^, Yaming Liu ^a, b^, Ying Li ^c^, Zhaoling Sun ^d^, Chao Zhi ^a, b, *^

Ning Niu and Lingjie Yu contributed equally to this work.

^a^ School of Textile Science and Engineering, Xi’an Polytechnic University, Xi’an, Shaanxi 710048, China

^b^ Key Laboratory of Functional Textile Material and Product, Ministry of Education, Xi’an Polytechnic University, Xi’an, Shaanxi 710048, China

^c^ Shaanxi Textile Science Research Institute Co., Ltd., Xi’an, Shaanxi 710016, China

^d^ College of Textile Science and Engineering, Jiangnan University, Wuxi, Jiangsu 214122, China

^*^ Corresponding author.


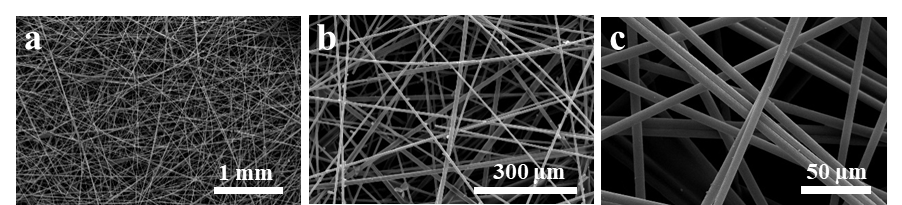


**Figure S1.** SEM images of CFF: a) Magnification of 100×. b) Magnification of 500×. c) Magnification of 2000×.


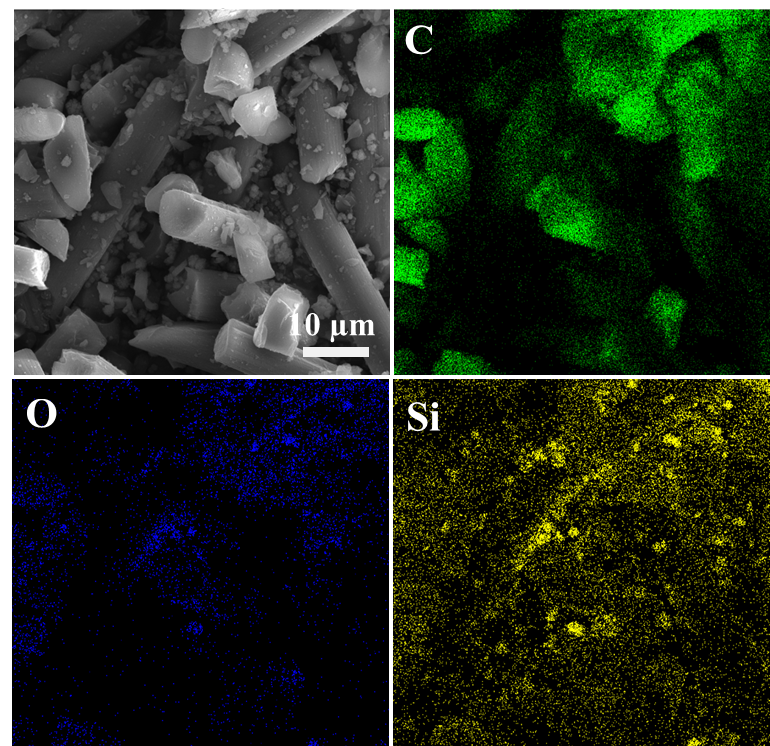


**Figure S2.** Mapping result of PDMS-CFs-CFF.


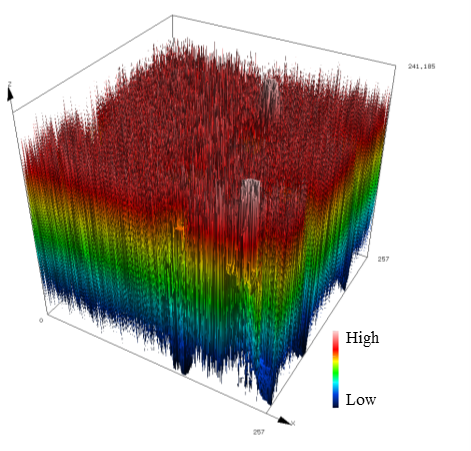


**Figure S3.** Confocal microscopy image of PDMS-CFs-CFF.


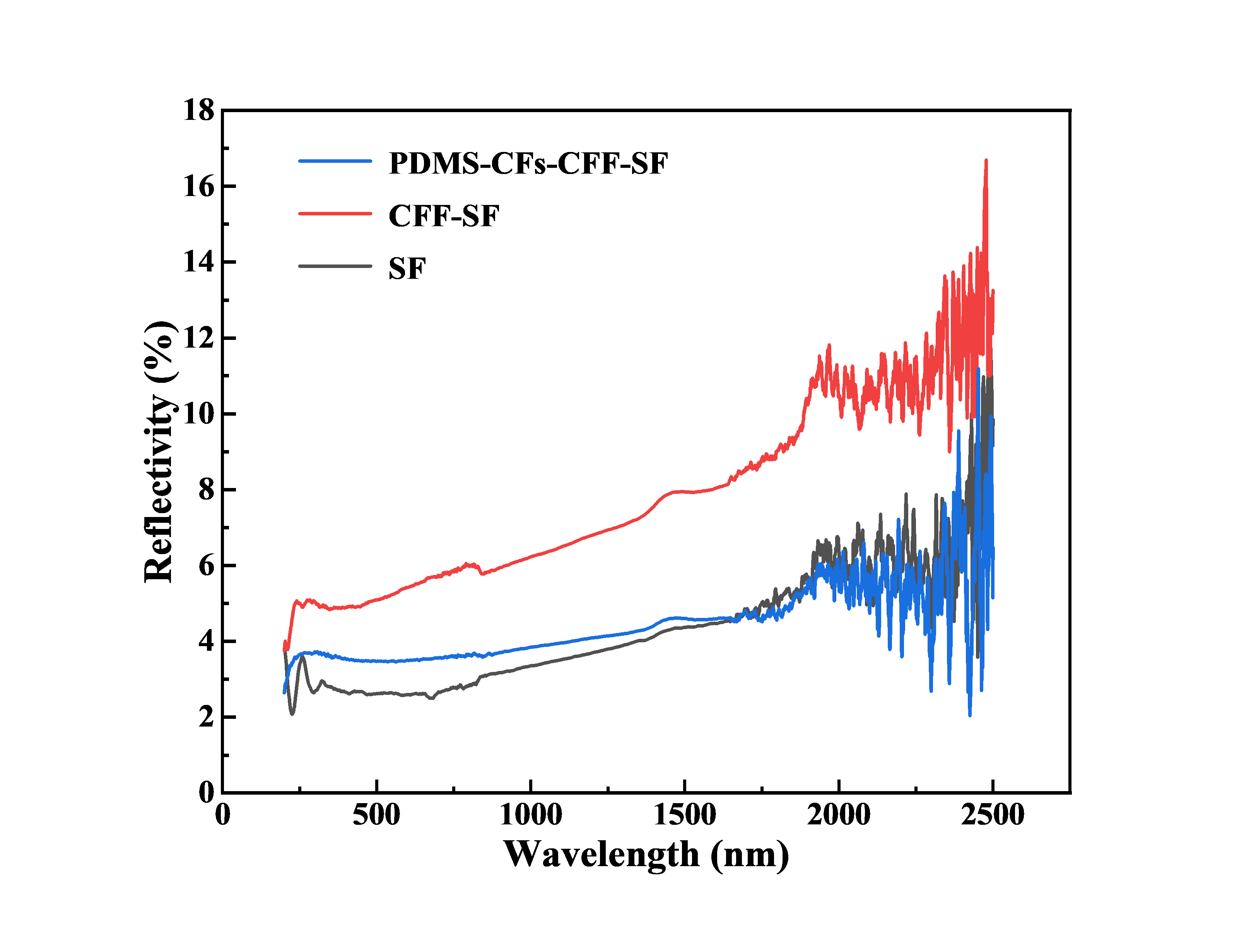


**Figure S4.** Light reflectance image of PDMS-CFs-CFF-SF, CFF-SF, and SF.


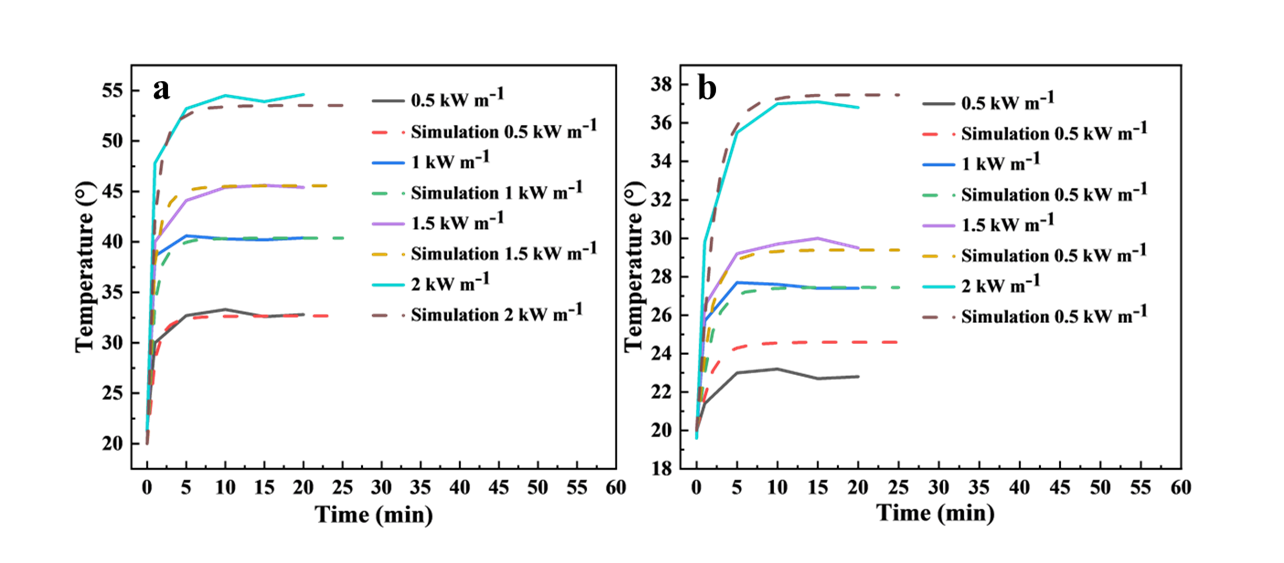


**Figure S5.** Schematic diagram of temperature change over time and simulation curves for wet PDMS-CFs-CFF-SF: a) Upper interface; b) Lower interface.


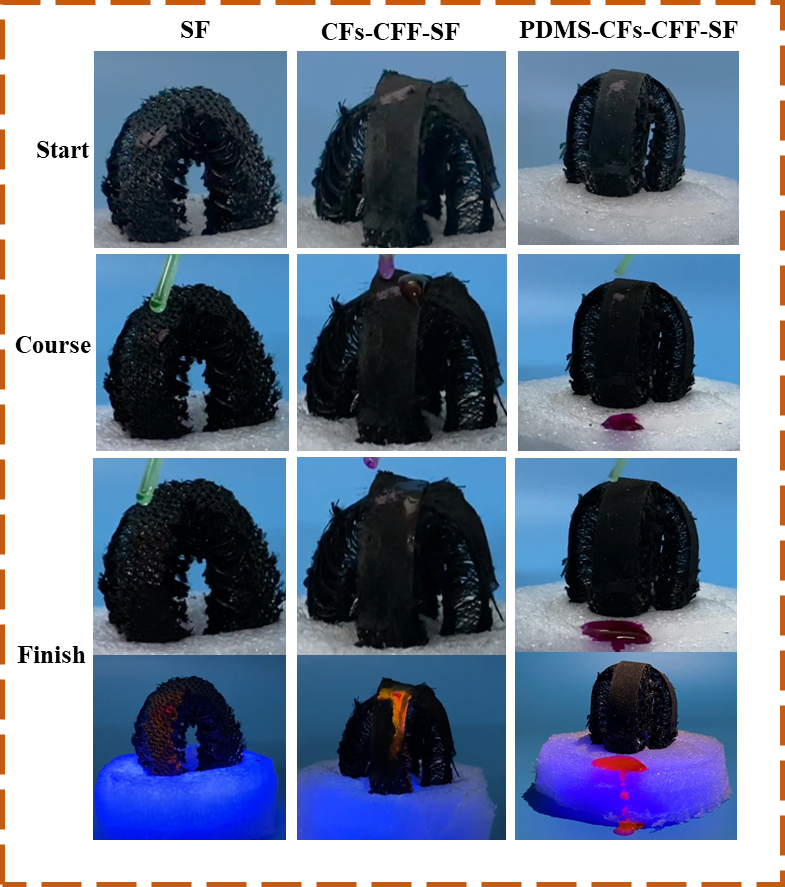


**Figure S6.** Self-cleaning experimental images of SF, CFs-CFF-SF, and PDMS-CFs-CFF-SF.


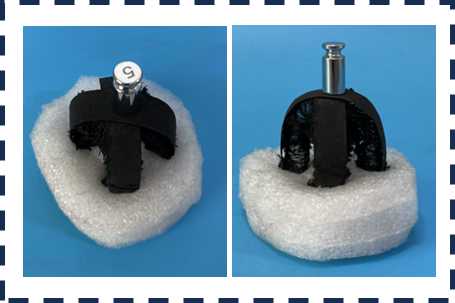


**Figure S7.** Images of PDMS-CFs-CFF-SF with a 5 g weight placed on it.


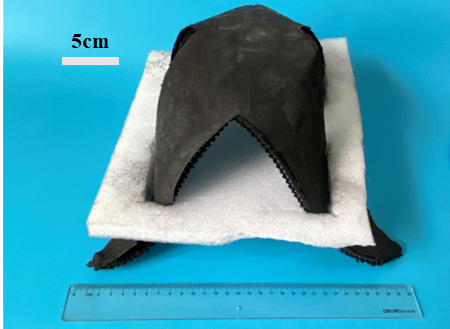


**Figure S8.** Image of large-size sample.

**Table S1**. The cost of the PDMS-CFs-CFF-SF.

| **Material** | **Unit price** | **Amount** | **Cost** |
| --- | --- | --- | --- |
| **Spacer fabrics** | 6.3 $ m^-2^ | 1 m^2^ | 6.3 $ |
| **Carbon fiber felt** | 3.8 $ m^-2^ | 1 m^2^ | 3.8 $ |
| **Carbon fibers** | 7.7 $ m^-2^ | 400 g | 3.1 $ |
|  |  | **Total** | 13.2 $ |


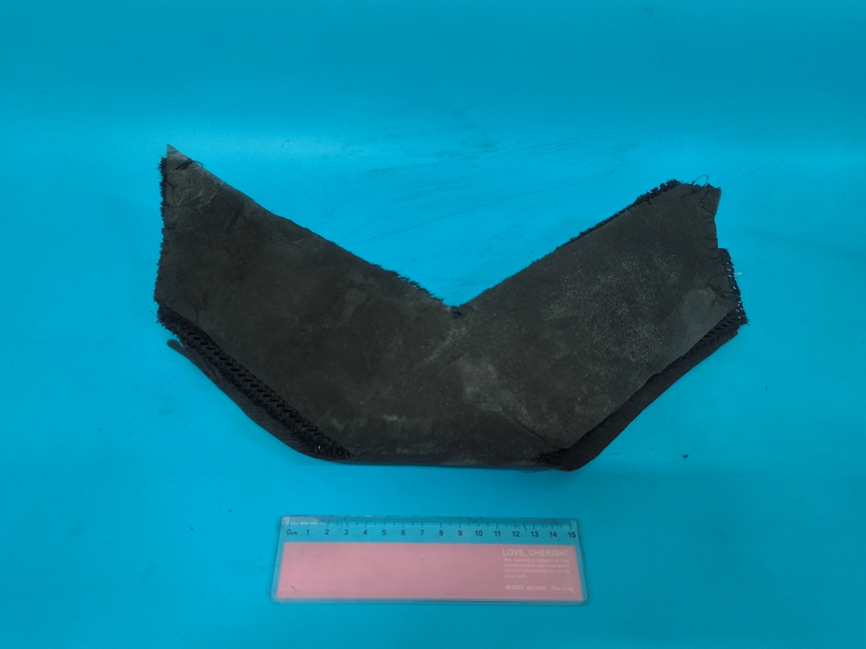


**Figure S9.** Folding image of large-size sample.


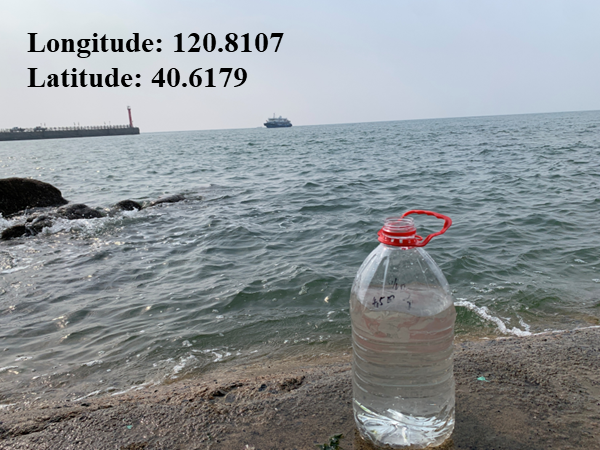


**Figure S10.** The water collection photograph of the Bohai Sea.


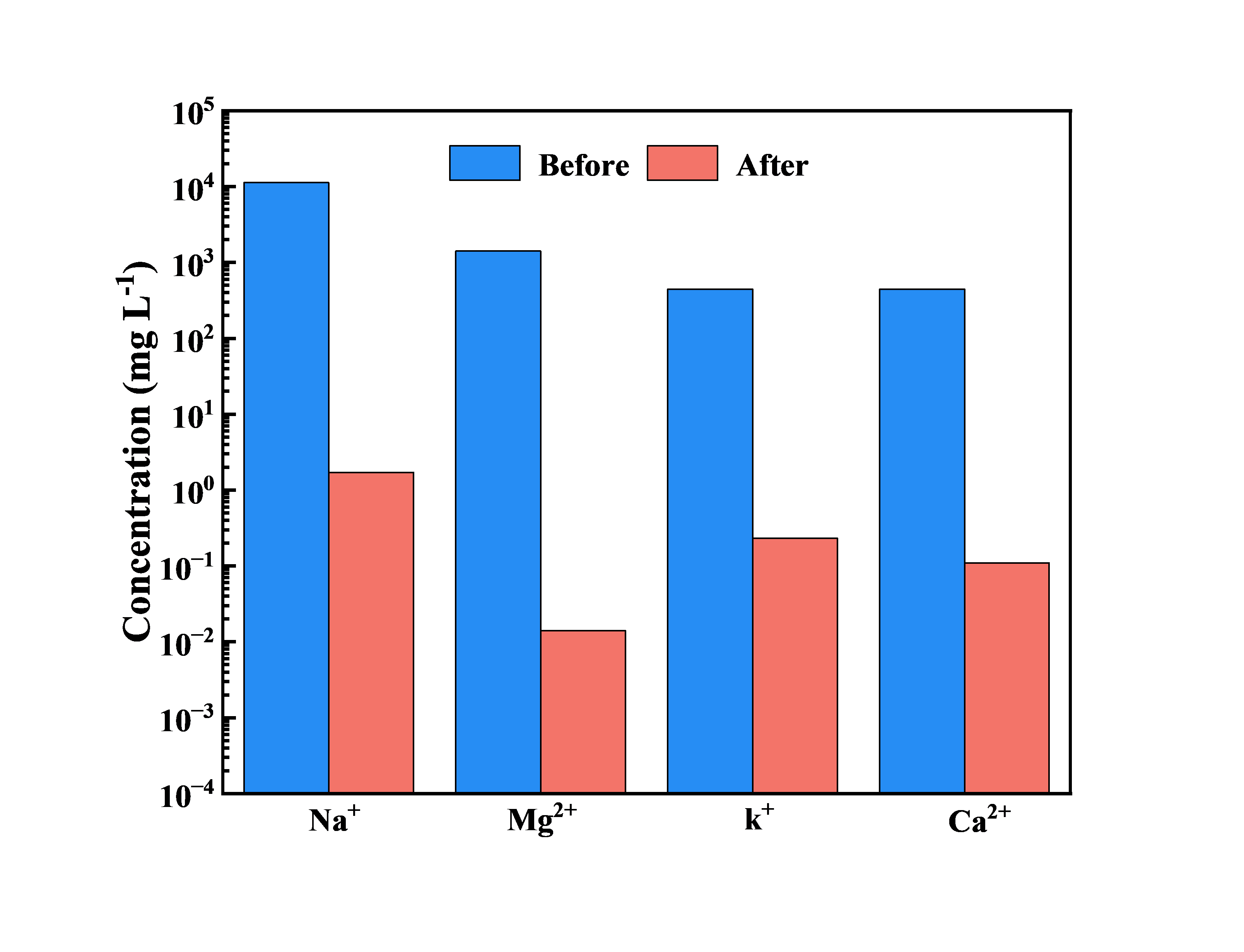


**Figure S11.** The concentration changes of four major seawater ions in artificial seawater before and after desalination.


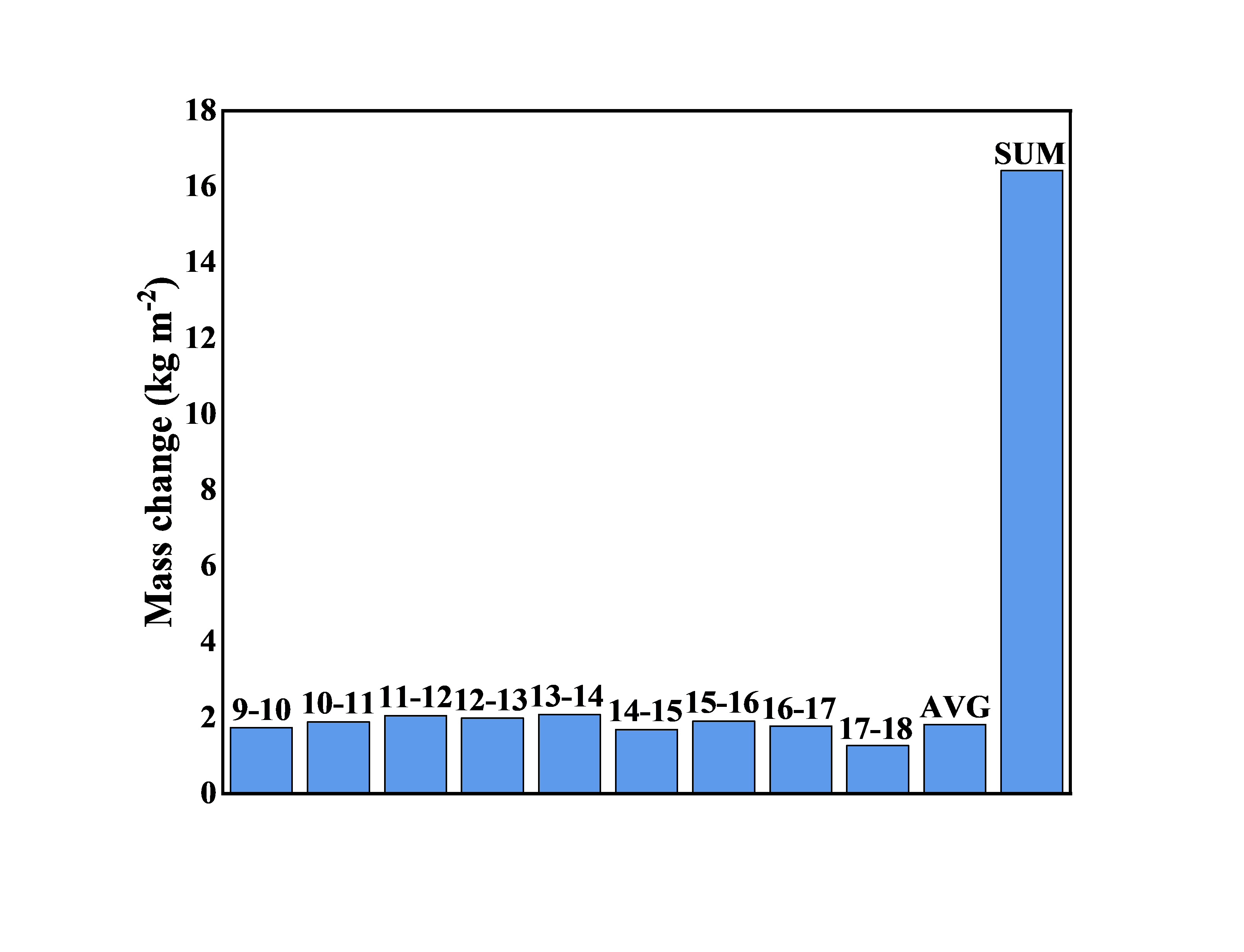


**Figure S12.** Bar chart of hourly evaporation amount, average evaporation amount, and total evaporation amount.


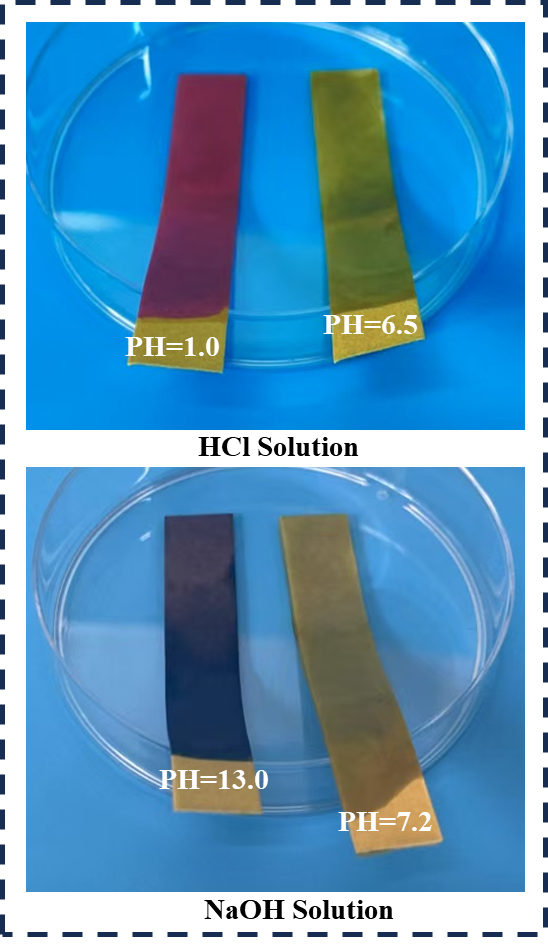


**Figure S13.** Comparison of pH values of HCl and NaOH solutions before and after purification.


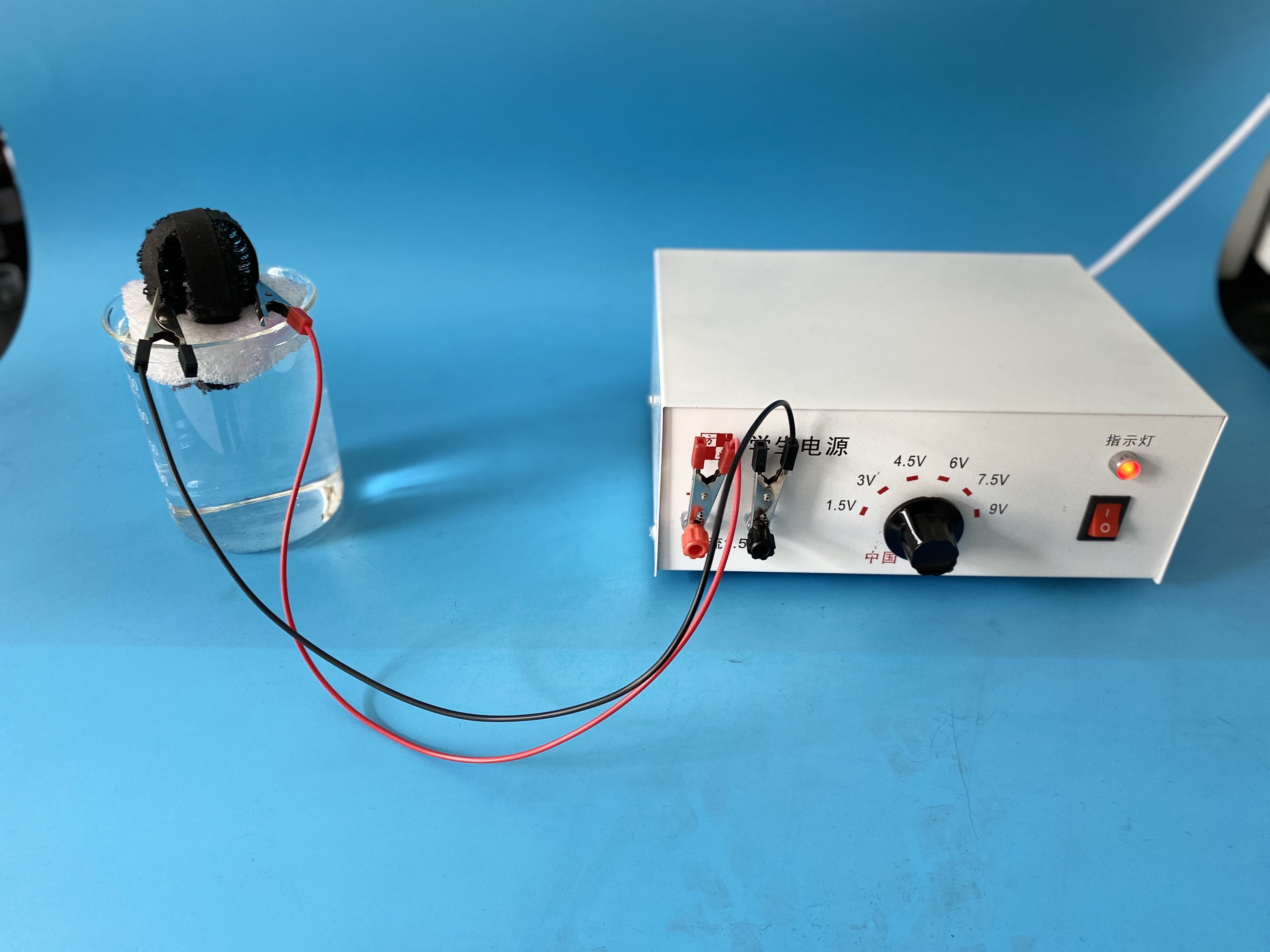


**Figure S14.** Image of PDMS-CFs-CFF-SF evaporating with an external power supply.


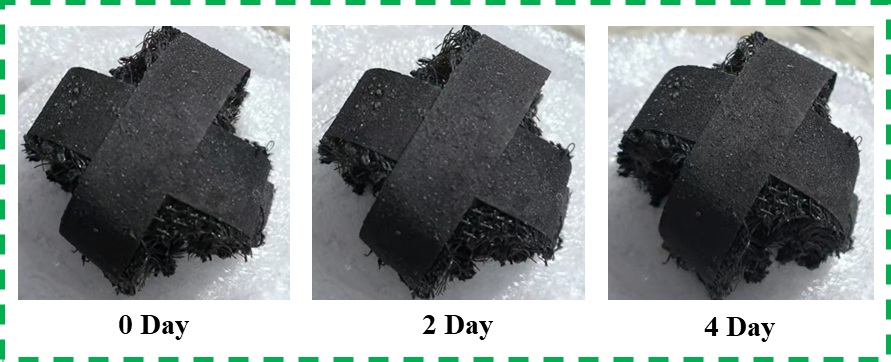


**Figure S15.** Photographs of PDMS-CFs-CFF-SF during solar-driven desalination in Bohai Sea water.


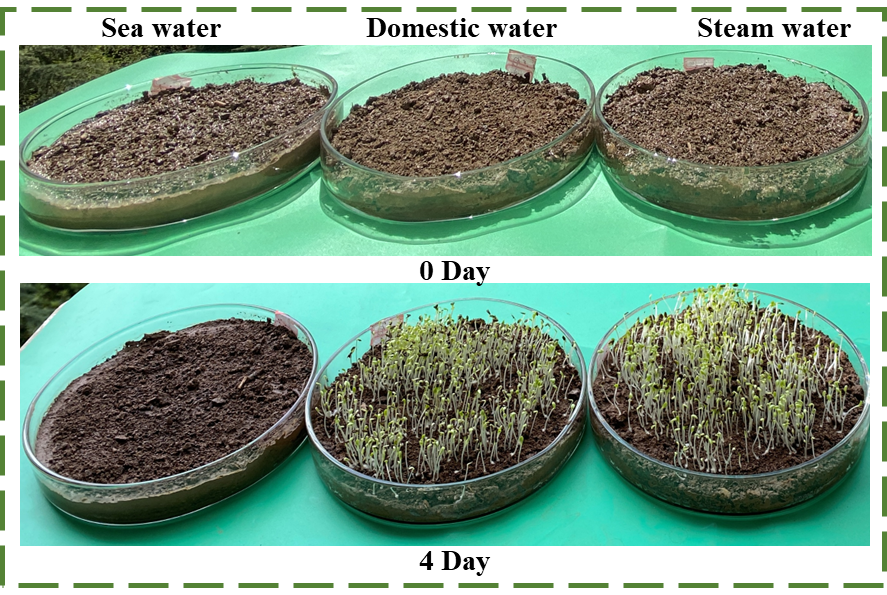


**Figure S16.** Growth images of lettuce seedlings irrigated with different water sources.

**Table S2.** The method for threading yarn of spacer fabric (SF).

| **Guide bar comb** | **Method for threading yarn ^a)^** |
| --- | --- |
| GB1 | full threading |
| GB2 | full threading |
| GB3 | full threading |
| GB4 | full threading |
| GB5 | two emptying and two threading |
| GB6 | two emptying and two threading |

a) GB stands for guide bar combs; Full threading means that each of the inverted pins of the warp knitting machine is threaded by yarn; Two emptying and two threading means that the yarn skips two inverted pins for every two it threads through.

**Table S3.** The chain block motion of SF.

| **Chain block** | **Lapping code ^b)^** |
| --- | --- |
| L1 | 2222 0000 *6 |
| L2 | 0111 1000 *6 |
| L3 | 1023 4532 *6 |
| L4 | 1000 0111 *6 |
| L5 | 4544 3233 4533 1011 2322 1022 *2 |
| L6 | 1001 2322 1022 4544 3233 4533 *2 |

b) L stands for chain block, * stands for the number of cycles.

**Table S4.** Specifications of SF.

| **Upper layer structure** | **Bottom layer structure** | **Polyester multifilament for surface layer yarn** | | **Spacer yarn diameter (mm)** | **Thickness (mm)** | **Area density**  **(g m^-2^ )** |
| --- | --- | --- | --- | --- | --- | --- |
| hexagonal mesh | Chain +inlay | | 300D/96F^a)^ | 0.16 | 7 | 680 |

a) D stands for denier, defined as the mass in grams per 9000 m; F stands for filament, indicating the number of filaments contained in a multifilament yarn.


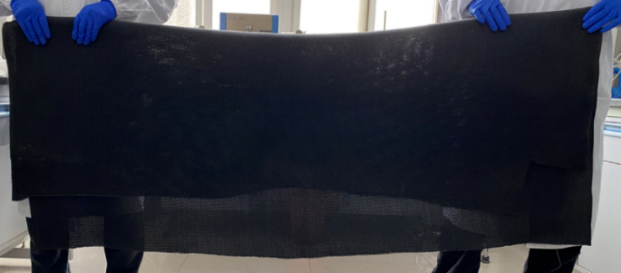


**Figure S17.** Optical image of SF.


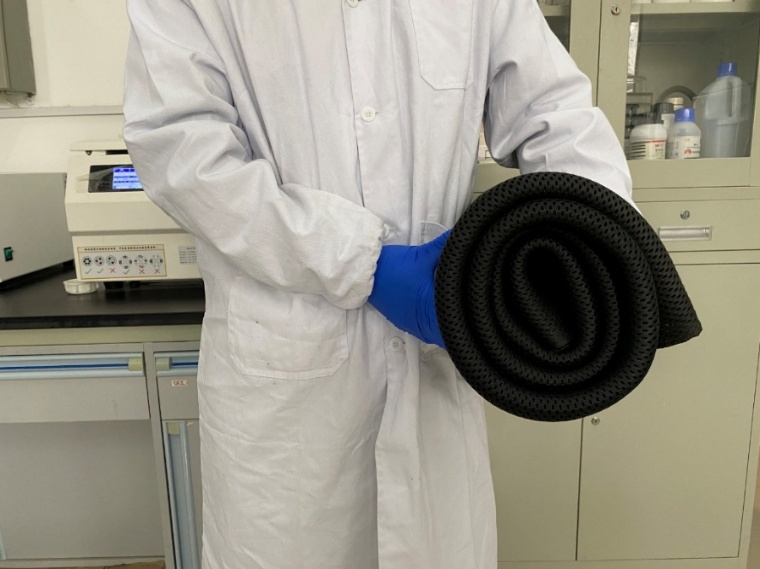


**Figure S18.** Curled image of SF.

**Calculation formula**

The ∆*H* of pure water at different temperature can be described by Equation:

 (1)

Where *C*_1_=2500.304, *C*_2_= -2.2521025, *C*_3_= -0.021465847, *C*_4_= 3.1750136×10^-4^, *C*_5_= -2.8607959×10^-5^ are constants, and *T* is temperature (°C).

To accurately estimate the low-temperature evaporation enthalpy of water on the surface of PDMS-CFs-CFF-SF, constant-temperature evaporation experiments were conducted at 25 °C and RH=50% in a controlled environment for pure water, single-interface evaporator, PDMS-CFs-CFF-SF, N-PDMS-CFs-CFF-SF. The experiments recorded the sample mass loss and calculated the hourly evaporation rate. Assuming that the evaporation of water at 25 °C is solely driven by energy input from the surrounding environment (*U*_in_), the relationship between the rate of mass change (*V*) and the evaporation enthalpy of water (∆*H*) is as follows:

 (2) Where ∆*H* and ∆*H*_1_ respectively denote the evaporation enthalpy of pure water and water on the surface of the material, and *V*and *V*_1_ represent the evaporation rates of pure water and water on the sample surface, respectively, under the same environmental conditions.

The evaporation enthalpies of single-interface evaporator, PDMS-CFs-CFF-SF, N-PDMS-CFs-CFF-SF were experimentally obtained and measured as 2175.48 kJ kg^-1^ (single-interface evaporator, PDMS-CFs-CFF-SF, N-PDMS-CFs-CFF-SF share the same chemical composition; therefore, their evaporation enthalpy should theoretically be identical), respectively. Substituting the solar irradiance *Q*_i_ = 1.0 kW m^-2^ into the formula, the calculated evaporation efficiencies for the single-interface evaporator, PDMS-CFs-CFF-SF, N-PDMS-CFs-CFF-SF were 97.90%, 129.32%, 78.56% ,respectively.

**Table S5.** Detailed parameters of evaporation efficiency calculation.

|  | Evaporation rate under 1 sun  (kg m^-2^ h^-1^) | | | | Evaporation rate in darkness  (kg m^-2^ h^-1^) | *V*  (kg m^-2^ h^-1^) | | ∆*H*  (kJ kg^-1^) |
| --- | --- | --- | --- | --- | --- | --- | --- | --- |
| Pure water | | 0.45 | | 0.14 | | | 0.31 | 2441.86 |
| Single-interface evaporator | | | 2.1 | 0.48 | | | 1.62 | 2175.48 |
| PDMS-CFs-CFF-SF | | 3.14 | | 1 | | | 2.14 | 2175.48 |
| N-PDMS-CFs-CFF-SF | | 1.65 | | 0.62 | | | 1.3 | 2175.48 |


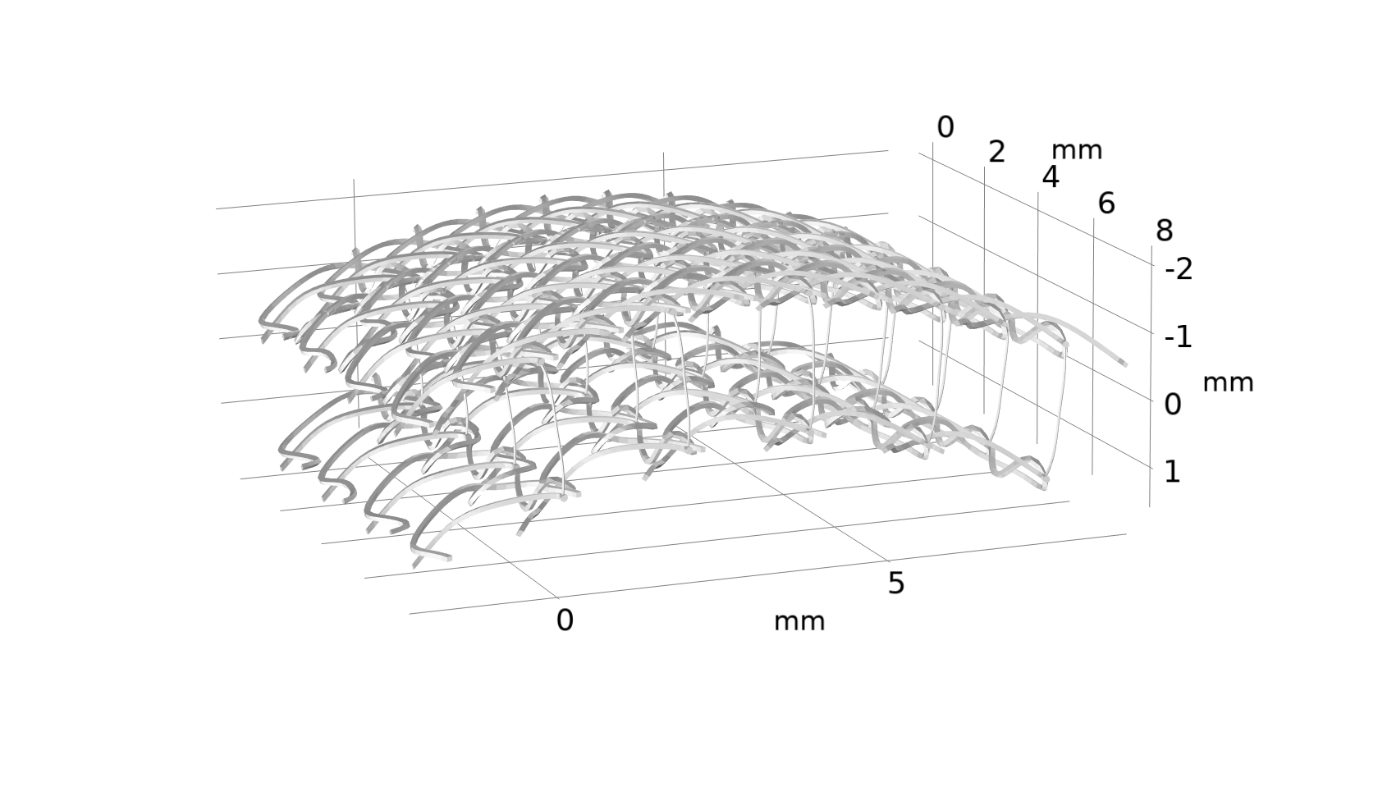


**Figure S19.** Finite element models of PDMS-CFs-CFF-SF.


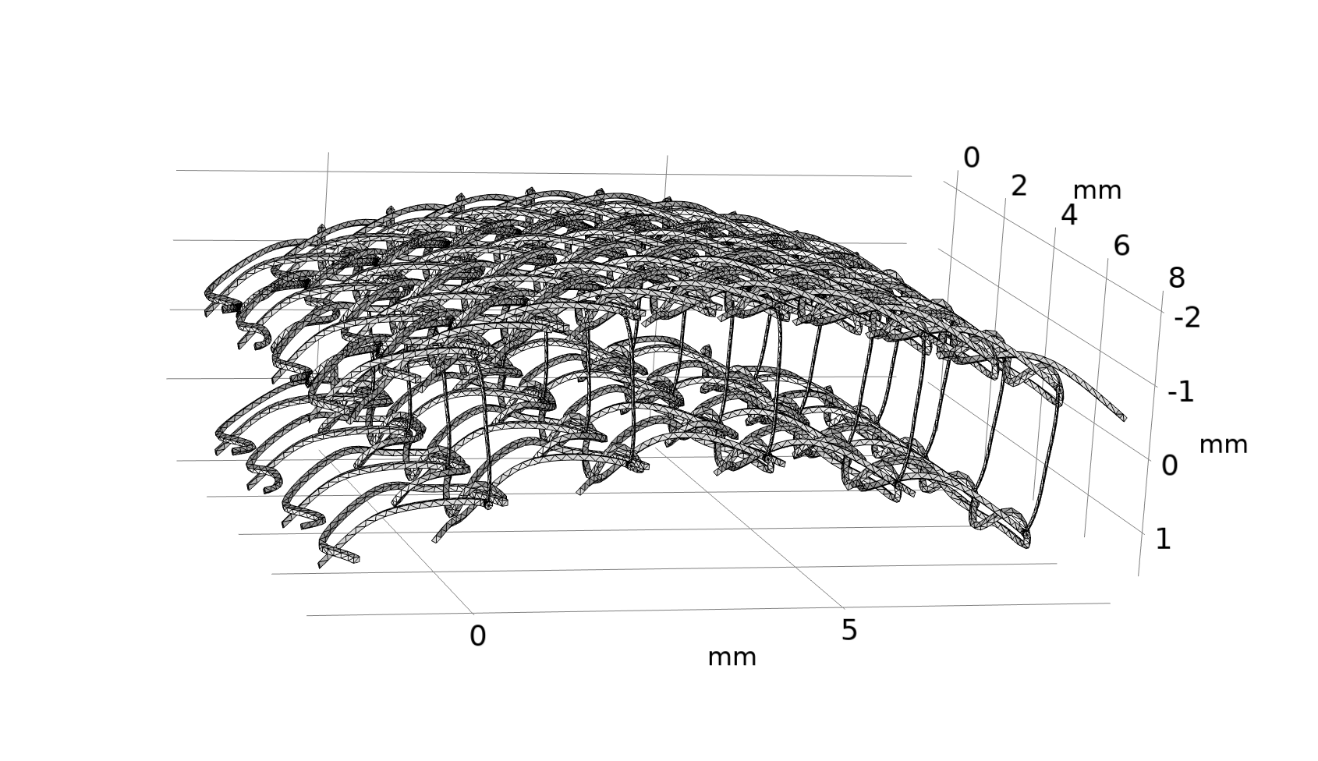


**Figure S20.** Mesh division of PDMS-CFs-CFF-SF.
